# A non-invasive nanoparticles for multimodal imaging of ischemic myocardium in rats

**DOI:** 10.1186/s12951-021-00822-7

**Published:** 2021-03-22

**Authors:** Xiajing Chen, Yanan Zhang, Hui Zhang, Liang Zhang, Lingjuan Liu, Yang Cao, Haitao Ran, Jie Tian

**Affiliations:** 1 Department of Cardiology, Ministry of Education Key Laboratory of Child Development and Disorders, National Clinical Research Center for Child Health and Disorders (Chongqing), China International Science and Technology Cooperation Base of Child Development and Critical Disorders, Children’s Hospital of Chongqing Medical University, Chongqing, 400014 People’s Republic of China; 2 Chongqing Key Laboratory of Pediatrics, Children’s Hospital of Chongqing Medical University, Chongqing, 400014 People’s Republic of China; 3 Chongqing Key Laboratory of Ultrasound Molecular Imaging & Department of Ultrasound, The Second Affiliated Hospital of Chongqing Medical University, Chongqing, 400010 People’s Republic of China

**Keywords:** Ischemic myocardium, Nanoparticles, Non-invasive, Multimodal imaging

## Abstract

**Background:**

Ischemic heart disease (IHD) is the leading cause of morbidity and mortality worldwide, and imposes a serious economic load. Thus, it is crucial to perform a timely and accurate diagnosis and monitoring in the early stage of myocardial ischemia. Currently, nanoparticles (NPs) have emerged as promising tools for multimodal imaging, because of their advantages of non-invasion, high-safety, and real-time dynamic imaging, providing valuable information for the diagnosis of heart diseases.

**Results:**

In this study, we prepared a targeted nanoprobe (termed IMTP-Fe_3_O_4_-PFH NPs) with enhanced ultrasound (US), photoacoustic (PA), and magnetic resonance (MR) performance for direct and non-invasive visual imaging of ischemic myocardium in a rat model. This successfully designed nanoprobe had excellent properties such as nanoscale size, good stability, phase transformation by acoustic droplet vaporization (ADV), and favorable safety profile. Besides, it realized obvious targeting performance toward hypoxia-injured cells as well as model rat hearts. After injection of NPs through the tail vein of model rats, in vivo imaging results showed a significantly enhanced US/PA/MR signal, well indicating the remarkable feasibility of nanoprobe to distinguish the ischemic myocardium.

**Conclusions:**

IMTP-Fe_3_O_4_-PFH NPs may be a promising nanoplatform for early detection of ischemic myocardium and targeted treatment under visualization for the future.

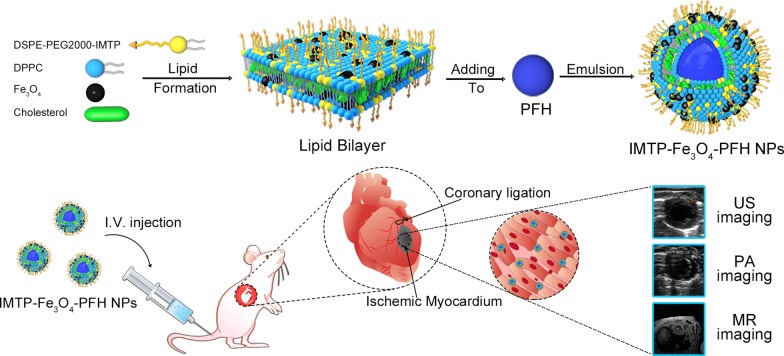

**Supplementary Information:**

The online version contains supplementary material available at 10.1186/s12951-021-00822-7.

## Introduction

Cardiovascular diseases (CVDs) are by far the leading cause of death in the world and are expected to account for > 22.2 million deaths by 2030 [1]. Despite considerable improvements in medical care, according to the American Heart Association in 2016, the estimated total costs of CVDs are expected to reach $1.1 trillion in 2035, the burden of which is quite heavy [1, 2]. Ischemic heart disease (IHD), especially caused by coronary artery obstruction or stenosis, is a major contributor of CVDs, in which persistent ischemia causes irreversible myocardial damage, resulting in profound myocardial cell death, and permanent loss of contractile function [3]. Therefore, how to achieve a timely and accurate diagnosis and monitor in the early stage of ischemia is crucial, and cardiac imaging plays a pivotal role in this process.

In recent years, non-invasive cardiac imaging has continued to evolve rapidly, which is advocated in patients with suspected ischemia before making hemodynamic reconstruction decisions [4–6]. Conventional non-invasive imaging tools mainly include ultrasonography, magnetic resonance (MR) imaging, single-photon emission computed tomography (SPECT), and positron emission tomography (PET) [7, 8]. Currently, ultrasonography is still the most widely used clinical imaging modalities for IHD, not only because it allows for a combined assessment of cardiac structure and function, particularly myocardial thickness and motion, but also because it can be easily used at the bedside [3, 9]. When the onset exceeds 20% of the transmural myocardial thickness, echocardiography almost immediately reveals ischemia-induced regional wall motion abnormalities [10, 11]. Furthermore, the high-resolution and high-contrast properties of cardiac magnetic resonance (CMR) has the same ability as echocardiography in the diagnosis of ischemic myocardium and can even predict prognosis by assessment markers of myocardial injuries, such as myocardial edema, intramyocardial hemorrhage, and infarct size [12]. However, every single modality of imaging has its own disadvantages, and no one can offer a perfect solution to the detection challenge. For instance, echocardiography lacks reproducibility and varies in image quality [13]; MRI requires long scanning time and is contraindicated in patients with a pacemaker [14]; SPECT and PET both use ionizing radiation and suffer from low spatial resolution [15–17].

Over recent decades, owing to the tremendous development of new material science and nanotechnology, multimodal imaging probes that integrate multiple imaging modalities into a complementary agent provide a new path for diagnosis [18–20]. Microbubbles have already been used clinically as ultrasound contrast agents to enhance imaging. However, defects such as the large size and the short lifespan of these gas-filled microbubbles restrict their imaging effects [21, 22]. Fortunately, nanoparticles (NPs) with perfluorocarbon via acoustic droplet vaporization (ADV) achieve great solutions to these problems [23, 24]. Liquid perfluorocarbon, such as perfluorohexane (PFH), pefluoropentane (PFP) and so on, in the core of NPs often stabilized with albumin, lipids, or polymers, vaporizes to gas phase upon activation by ultrasound (US) energy, providing a long-circulating, triggerable contrast agent [25]. Besides, low intensity focused ultrasound (LIFU) pioneered in our lab with limited ultrasound energy, can precisely focus on the target tissue, further reduce damage to the surrounding normal tissues [26–28].

Superparamagnetic iron oxide nanoparticles (SPIONs) as a negative contrast agent in T_2_-weighted imaging (T_2_-WI), are already used both for medical research and clinical diagnosis [29]. Moreover, our group previously demonstrated that SPIONPs also could be employed in photoacoustic (PA) imaging [26, 30–32]. PA imaging is an emerging diagnostic method with non-invasive and non-ionizing properties, availing the benefits of optical resolution, demonstrated promising potential in preclinical and clinical applications [33, 34]. The MRI − PA imaging combination can share mutual benefits, and overcome their shortages.

Over the past several decades, liposomes have been used in a variety of applications, as delivery vehicles for drugs, genetic material, and imaging agents [35, 36]. On the one hand, the ease with which lipid nanoparticles can integrate a variety of imaging agents, from fluorescent molecules to chelated metals and nanocrystals, is the most important reason for their popularity [20]. On the other hand, liposomal NPs are based on natural phospholipids, which greatly enhances their biocompatibility and safety. Current liposome-based research is more focused on tumors, while CVDs treatment and diagnosis are susceptible to ineffectiveness due to high shear stress washout and short time retention [37, 38]. Targeting liposomes specifically to the myocardium may alleviate these problems, consequently improving the delivery efficiency. Ischemic myocardium-targeted peptide (IMTP) is a cyclic 9 amino acid sequence (CSTSMLKAC) screened by in vivo phage display [39]. Many studies have proved that this peptide can home preferentially to cardiomyocytes in the ischemic myocardium, used as an active targeting marker [40–43].

**Scheme 1:**
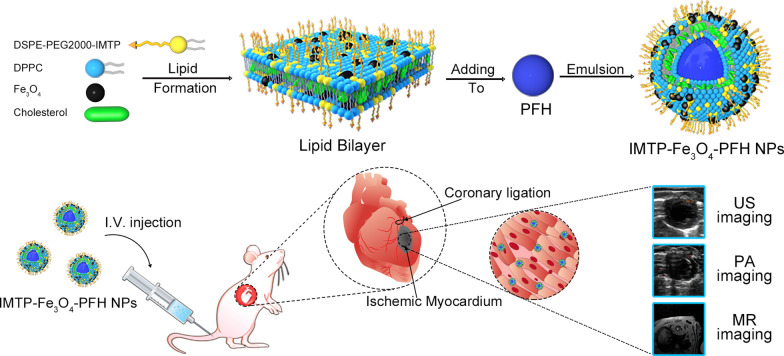
**a** Synthetic procedure of IMTP-Fe_3_O_4_-PFH NPs and **b** illustration of IMTP-Fe_3_O_4_-PFH NPs enhanced multimodal imaging for ischemic myocardium in rats

Inspired by the research described above, we herein proposed a nanoplatform as a non-invasive, and ischemic myocardium-targeted probe (termed IMTP-Fe_3_O_4_-PFH NPs) for multimodal imaging (US/PA/MR imaging) in rats (Scheme 1). PFH had been employed as phase-transformation material, encapsulated in liposomes. After the LIFU irradiation, the ADV-generated microbubbles could further enhance US imaging. With the help of iron oxide nanoparticles (Fe_3_O_4_), these NPs were applied for PA and MR imaging. Moreover, IMTP had been conjugated on the NPs surface for active targeting. As such, a probe with nanoscale size, high affinity for ischemic cardiomyocytes, good biocompatibility, and obviously enhanced multimodal imaging both in vitro and in vivo was expected, which may lay the foundation for the next generation contrast agent to identify ischemic myocardium and even visualize its treatment in future.

## Materials and methods

### Materials

The lipid components used in the formation of NPs included 1,2-Dipalmitoyl-sn-glycero-3-phosphatidylcholine (DPPC), 1,2-Distearoyl-sn-glycero-3-phosphoethanolamine-N- [methoxy (polyethylene glycol)-2000] (DSPE-PEG2000), and cholesterol (Chol), which were all purchased from Corden Pharma Inc (Liestal, Switzerland). IMTP (CSTSMLKAC, cyclic form, Mw = 943.2 Da) and DSPE-PEG2000-IMTP were synthesized and purified by Xi’an ruixi Biological Technology Co., Ltd (Shanxi, China). Iron oxide nanoparticles (10 nm) with oleic acid (OA- Fe_3_O_4_) were purchased from Ocean Nano Technology Co., Ltd (Springdale, AR, USA). PFH was obtained from J&K Scientific Ltd (Beijing, China). 1,1′-Dioctadecyl-3,3,3′,3′-tetramethylindocarbocyanine perchlorate (DiI) was provided from Beyotime Biotechnology (Shanghai, China). 4′, 6-Diamidino-2-phenylindole (DAPI) and 1,1′-Dioctadecyl-3,3,3′,3′-tetramethylindotricarbocyanine iodide (DiR) were purchased from Sigma-Aldrich (Saint Louis, MO, USA). Agarose was received from Invitrogen (Carlsbad, California, USA). Dulbecco’s modified Eagle’s medium (DMEM), fetal bovine serum (FBS), and trypsin were purchased from Gibco Co. (Carlsbad, CA, USA). Penicillin–streptomycin was obtained from Boster Biological Technology Co., Ltd (Wuhan, China). Cell Counting Kit 8 (CCK-8) was provided by Dojindo Molecular Technology (Tokyo, Japan). Hydrogen peroxide (H_2_O_2_), trichloromethane (CHCl_3_), and other analytical grade reagents were purchased from Chongqing Chuandong chemicals (Chongqing, China).

### Synthesis of NPs

10 mg of DPPC, 4 mg of DSPE-PEG2000-IMTP, 3 mg of cholesterol, and 200 ul OA-Fe_3_O_4_ (5 mg/ml) were dissolved into 5 ml of CHCl_3_, and then the solution was transferred to a round flask to form lipid films by rotary evaporation (Yarong Inc, Shanghai, China). Two hours later, the lipid film that appeared at the bottom of the flask was hydrated by 4 ml of double-distilled water. After adding 200 μl of PFH, the suspension was emulsified in an ice-water bath using a sonicator (Sonics & Materials Inc., USA) at 125 W for 8 min (5 s on and 5 s off). To remove free lipids and excess reactants, the nano-emulsion was centrifuged at 8,000 rpm for 5 min at 4 ℃ and repeated thrice. Finally, IMTP-Fe_3_O_4_-PFH NPs were obtained and then stored at 4 ℃ for later use.

The preparation method of non-targeted NPs (Fe_3_O_4_-PFH NPs) was the same as described above, except that DSPE-PEG2000 was used instead of DSPE-PEG2000-IMTP. Additionally, IMTP-PFH NPs were acquired as the ferric blank control without OA-Fe_3_O_4_ loading, and similarly, PFH was not added as IMTP-Fe_3_O_4_ NPs.

To prepare various fluorescent NPs, the fluorescent dye DiI or DiR (5 μl) was added to the lipid solution for rotary evaporation together, and tinfoil was used to prevent light exposure.

### Characterization

The size, morphology, and dispersion of the IMTP-Fe_3_O_4_-PFH NPs were observed by the light microscope (Nikon, Japan) after dilution 100 times. A transmission electron microscope (TEM) (H-7600; Hitachi, Tokyo, Japan) was used to further ratify the IMTP-Fe_3_O_4_-PFH NPs morphology. The mean particle size and zeta potential of the different NPs were detected by Zetasizer NANO ZS system (Malvern Instruments Ltd., Malvern, UK). The concentration of Fe was measured with an atomic absorption spectrometer (Hitachi model Z-5000, Hitachi Ltd., Tokyo, Japan). The encapsulation efficiency and loading capacity of Fe_3_O_4_ were calculated by the following equation: Encapsulation efficiency (%) = mass of Fe_3_O_4_/total Fe_3_O_4_ × 100%; Loading content (%) = mass of Fe_3_O_4_/total liposomes × 100%. To test the stability of IMTP-Fe_3_O_4_-PFH NPs, the size of NPs was measured at each indicated time point (0, 1, 3, 5, 7 d).

### Cell culture and animal model establishment

Rat myocardial cells (H9C2) purchased from Zhong Qiao Xin Zhou Biotechnology Co., Ltd (Shanghai, China), were cultured in DMEM supplemented with 10% FBS, penicillin (100 U/ml), and streptomycin (100 U/ml) at 37 °C in a humidified incubator under normal conditions (5% CO_2_, 21% O_2_ and 74% N_2_). After being cultured for 2–3 days, cells were trypsinzed and subcultured.

The hypoxia injury in cells was established by chemical reagent H_2_O_2_ or subjected to hypoxia conditions. In a subset of experiments, H9C2 cells were placed in a 37 °C airtight box saturated with 94% N_2_, 5% CO_2_, 1% O_2_ for different durations. Besides, the medium was changed to serum-free DEME. In another subset of experiments, cells were insulted with different concentrations of H_2_O_2_ for 24 h. Through cell morphology observation and CCK-8 detection of cell vitality, the most suitable conditions were selected for subsequent experiments.

Male Sprague–Dawley (SD) rats (with a bodyweight of 220 to 270 g) were maintained in the Center of Experimental Animals at Chongqing Medical University (Chongqing, China). Rats underwent ligation of the left anterior descending (LAD) coronary artery to induce acute myocardial ischemia. Briefly, the rats were anesthetized with 1% pentobarbital sodium (40 mg/kg, administered intraperitoneally), and ventilated via a rodent ventilator (Shanghai Alcott Biotech co., Ltd. China). The LAD was ligatured with a 6/0 suture. Successful occlusion of LAD was confirmed by the presence of S-T segment elevation on the electrocardiogram (ECG) (Guangzhou sanrui electronics co. Ltd, China) and a color change in ventricular from fresh red to pale. After confirmation of successful molding, the muscles and skin were sutured layer-by-layer and disinfected the incision. Postoperative echocardiography and histological examination after two weeks were used to further verify the success of modeling, so as to ensure the consistency of the model in subsequent experiments.

### In vitro cytotoxicity and in vivo biosafety assay of NPs

To evaluate the cytotoxicity of IMTP-Fe_3_O_4_-PFH NPs, H9C2 cells were seeded in a 96-well plate (0.5 × 10^5^ cells per well) and cultured for 24 h. DMEM without FBS was used to prepare different concentrations of IMTP-Fe_3_O_4_-PFH NPs (0.1, 0.2, 0.4, 0.8 and 1.6 mg/ml). After another 24 h co-incubation, cell viabilities were tested by the CCK-8 assay. Six replicates were set for each group.

To assess the in vivo toxicity of IMTP-Fe_3_O_4_-PFH NPs, twenty-five healthy SD rats were randomly divided into five groups as follows: control group, 1 d, 3 d, 7 d, and 14 d experimental group (n = 5 per group). Via tail vein injection, the control group was injected with saline (1 ml), and the experimental groups were all administrated with IMTP-Fe_3_O_4_-PFH NPs (1 ml, 5 mg/kg). During the whole period of the experiment, the abnormal reaction, death, and its occurrence time were observed and recorded. At different points in time, 3–5 ml of whole blood was collected from the heart, part of which was used for routine blood detection, and part of which was separated from serum for biochemical analysis. The routine blood test mainly contained red blood cell (RBC), white blood cell (WBC), platelet (PLT), hemoglobin (HGB), and mean corpuscular volume (MCV). The biochemical indexes included aspartate aminotransferase (AST), alanine aminotransferase (ALT), total bilirubin (TB), urea (UREA), creatinine (CREA), and lactate dehydrogenase (LDH). At the end of the experiment, rats were sacrificed and major organs (heart, liver, spleen, lung, and kidney) were taken for histological analysis with hematoxylin and eosin stain (H&E).

### In vitro targeting efficiency

According to the previous model establishment, respectively with low oxygen or H_2_O_2_ treatment, H9C2 cells were seeded in culture dishes specified for confocal laser scanning microscopy (CLSM) (Nikon, Japan) at a density of 1 × 10^4^ cells per dish. After 24 h, cells were divided into 4 groups: hypoxia + IMTP-Fe_3_O_4_-PFH NPs, hypoxia + Fe_3_O_4_-PFH NPs, H_2_O_2_ + IMTP-Fe_3_O_4_-PFH NPs, H_2_O_2_ + Fe_3_O_4_-PFH NPs. All groups were mixed with 100 μl DiI-labeled NPs (1 mg/ml) for 0.5 h, 1 h, 2 h, and 4 h respectively. After co-incubation, cells were washed thrice with PBS, fixed with 4% paraformaldehyde for 10 min, and then incubated with DAPI for 5 min. After that, the dishes were washed with PBS three times and sent for CLSM while protected from light.

### Ex vivo targeting ability and biodistribution

To study the targeting efficiency and biodistribution of the NPs, model rats were randomly divided into two groups (n = 3) and injected with DiR-labeled IMTP-Fe_3_O_4_-PFH NPs or Fe_3_O_4_-PFH NPs via the tail vein immediately after the operation. Ex vivo fluorescence imaging of the heart and other major organs, including lung, spleen, liver, and kidney were acquired at pre, 5, 10, 30 min, 1, 2, 4, and 8 h post-injection by a fluorescence system (CRi Inc, Woburn, MA, USA), and the relative fluorescence intensities were calculated.

### Phase transition and US imaging in vitro and in vivo

To determine whether IMTP-Fe_3_O_4_-PFH NPs could exhibit a phase transition behavior and whether PFH acted in this process, the IMTP-Fe_3_O_4_-PFH NPs and IMTP-Fe_3_O_4_ NPs were exposed to LIFU (1.0 MHz, focal length of 1.5 cm, duty cycle of 50%, pulse-wave mode, Chongqing Medical University, China) at different acoustic intensity (from 1 to 4 W/cm^2^) and different times (from 1 to 4 min), respectively. After irradiation, optical microscopy images were collected, and US images both in B-mode and contrast-enhanced ultrasound (CEUS) mode were evaluated by a US system (Esaote MyLab90, Florence, Italy) with a high-frequency linear array probe (LA523, frequency of 12 MHz, mechanical index of 0.06). Images before LIFU irradiation were used as controls. The echo intensities of regions of interest (ROI) were calculated by an ultrasound imaging software (DFY-II; Institute of Ultrasound Imaging, Chongqing, China) [44, 45].

For in vivo imaging, rats were divided randomly into 2 groups (n = 3): IMTP-Fe_3_O_4_-PFH NPs group, and Fe_3_O_4_-PFH NPs group. The chamber size, wall thickness, and amplitude of wall motion were assessed in the short-axis view. Each group of rats underwent ligation of LAD and were injected with NPs immediately as the baseline. 10 min after injection, all rats were irradiated by LIFU for 3 min (3 W/cm^2^, 1.0 MHz, focal length of 1.5 cm, duty cycle of 50%, pulse-wave mode). B-mode and CEUS images of pre-operation, baseline, after 10 min injection, and after LIFU irradiation were recorded. Besides, the intensities of ROI were compared.

### In vitro and in vivo PA imaging

PA imaging experiments were performed by a Vevo LAZR Photoacoustic Imaging System (VisualSonics Inc., Toronto, Canada). The maximum absorbance of NPs for PA imaging was scanned at different wavelengths ranging from 680 to 970 nm (interval = 5 nm), which was then used as a basis wavelength for subsequent imaging. The IMTP-Fe_3_O_4_-PFH NPs were diluted to a series of Fe concentration from 0.039 to 0.624 mg/ml and added in an agar gel mold to acquire the corresponding PA images. Finally, the PA intensities of each image were calculated using Vevo LAZR software.

As for PA imaging in vivo, the model rats were intravenously injected with IMTP-Fe_3_O_4_-PFH NPs and Fe_3_O_4_-PFH NPs, respectively. Then, PA images were acquired before the operation, immediately after injection (baseline), 10 min and 60 min after injection. Furthermore, the corresponding PA signal values were measured.

### In vitro and in vivo MR imaging

All MR experiments were performed conducted on a 7.0 T micro-MRI System (Bruker PharmaScan, Germany). The IMTP-Fe_3_O_4_-PFH NPs were dispersed in agar at different concentrations (0–0.32 mM) of Fe, and placed in 2 ml Eppendorf tubes for T_2_-WI using the following parameters: repetition time (TR) = 3000 ms, echo time (TE) = 9 ms, field of vision (FOV) = 45 × 40 mm, matrix = 256 × 256, slice thickness = 1.500 mm. The signal intensity of ROI was analyzed by RadiAnt DICOM Viewer and T_2_ relaxivities were calculated from the slope of the linear plots of r_2_ relaxation rates versus Fe concentration.

In vivo, MR imaging was conducted in model rats divided into 3 groups (n = 3), namely IMTP-Fe_3_O_4_-PFH NPs group, Fe_3_O_4_-PFH NPs group, and IMTP-PFH NPs group. Cardio-respiratory artifacts caused by cardiac pulsatility and respiration were reduced by using ECG and respiratory gating system. T_2_*-WI was run (TR = 40 ms, TE = 3.5 ms, flip angle = 40°, FOV = 40 × 40 mm, matrix = 192 × 192, slice thickness = 1 mm). Each image was processed by Matlab (2019) to get pseudo-coloring and the corresponding signal intensity was measured. After imaging, the hearts were harvested for Prussian blue & 3,3′-diaminobenzidine tetrahydrochloride (DAB) staining.

### Statistical analysis

All statistical analyses were performed with SPSS 20.0 software. Data were presented as mean ± standard deviation. One-way ANOVA and the Student’s *t*-test were utilized between two groups for statistical evaluation. P values < 0.05 were considered statistically significant.

## Results and discussion

### Synthesis and characterization of NPs

IMTP-Fe_3_O_4_-PFH NPs as an innovative non-invasive probe, in the composition of which, Fe_3_O_4_ acted as a contrast agent for PA/MR imaging, and PFH underwent a phase transition from liquid to gas to enhance US imaging after irradiation by LIFU. These NPs were constructed with a core/shell structure for enhanced colloidal stability and synthesized via a single-step emulsion method. The lipophilic OA-Fe_3_O_4_ was integrated into the lipid bilayer as the shell, and PFH was encapsulated as the core.

**Fig. 1:**
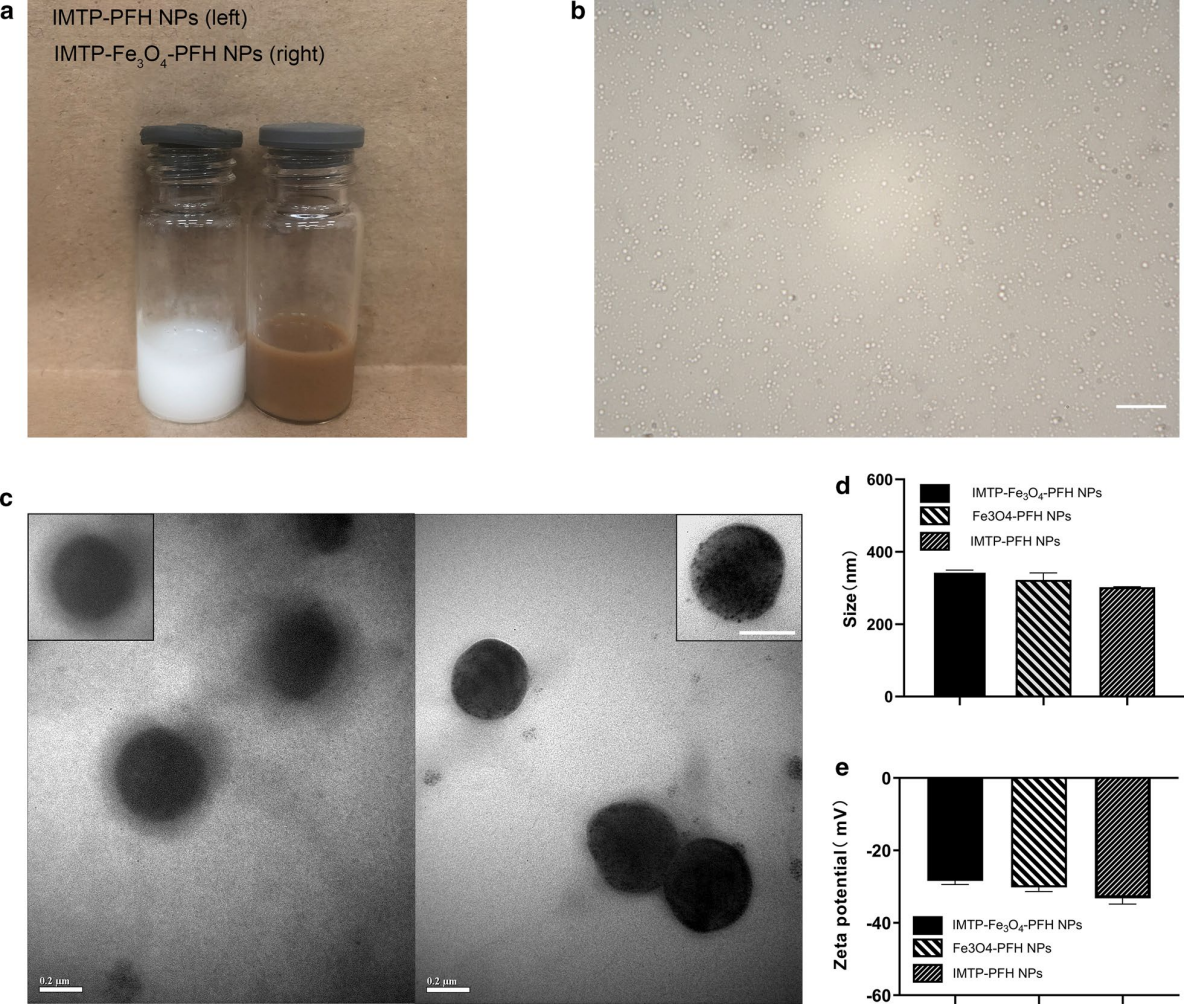
Characteristics of the NPs. **a** Photographs of IMTP-PFH NPs and IMTP-Fe_3_O_4_-PFH NPs. **b** Light microscope image of IMTP-Fe_3_O_4_-PFH NPs (scale bar: 10 μm). **c** Representative TEM images of IMTP-PFH NPs (left) and IMTP-Fe_3_O_4_-PFH NPs (right) (scale bar: 0.2 μm for both large and small view). **d** Size distribution of IMTP-Fe_3_O_4_-PFH NPs, Fe_3_O_4_-PFH NPs and IMTP-PFH NPs. **e** Zeta potentials of IMTP-Fe_3_O_4_-PFH NPs, Fe_3_O_4_-PFH NPs and IMTP-PFH NPs

As shown in Fig. 1a, after adding Fe_3_O_4_, the solution color changed from white to brown, which verified the success of Fe addition. Observed under a 100-fold oil lens of the microscope, NPs were spherical with a regular shape, uniform size, and even distribution (Fig. 1b). The ultrastructure of IMTP-Fe_3_O_4_-PFH NPs and IMTP-PFH NPs were observed by TEM, and the differences in their structure were obvious, where iron oxide particles were distributed in the former NPs, but none in the latter (Fig. 1c). The average diameters of IMTP-Fe_3_O_4_-PFH NPs, Fe_3_O_4_-PFH NPs, and IMTP-PFH NPs were 348.0 ± 1.2, 322.6 ± 19.3, and 302.2 ± 1.5 nm, respectively (Fig. 1d). The diameter of NPs with added iron oxide increased slightly compared to non-NPs, which further indicated the successful loading of Fe_3_O_4_ onto the NPs. Besides, the polydispersity index (PDI) of IMTP-Fe_3_O_4_-PFH NPs was 0.098 ± 0.026, which proved that these NPs were well dispersed with good homogeneity. The zeta potential of IMTP-Fe_3_O_4_-PFH NPs was -28.4 mV (Fig. 1e). During the seven-day observation period, no significant size change was observed, indicating the stability of NPs (Additional file 1: Figure S1). With the iron standard curves with good linear relationship by atomic absorption spectroscopy (R^2^ = 0.9954), thus detecting Fe content, the final calculation of encapsulation efficiency and loading capacity of Fe_3_O_4_ were 64.18 ± 0.26% and 9.44 ± 0.66%, respectively (Additional file 1: Figure S2).

### The safety profile of NPs

**Fig. 2:**
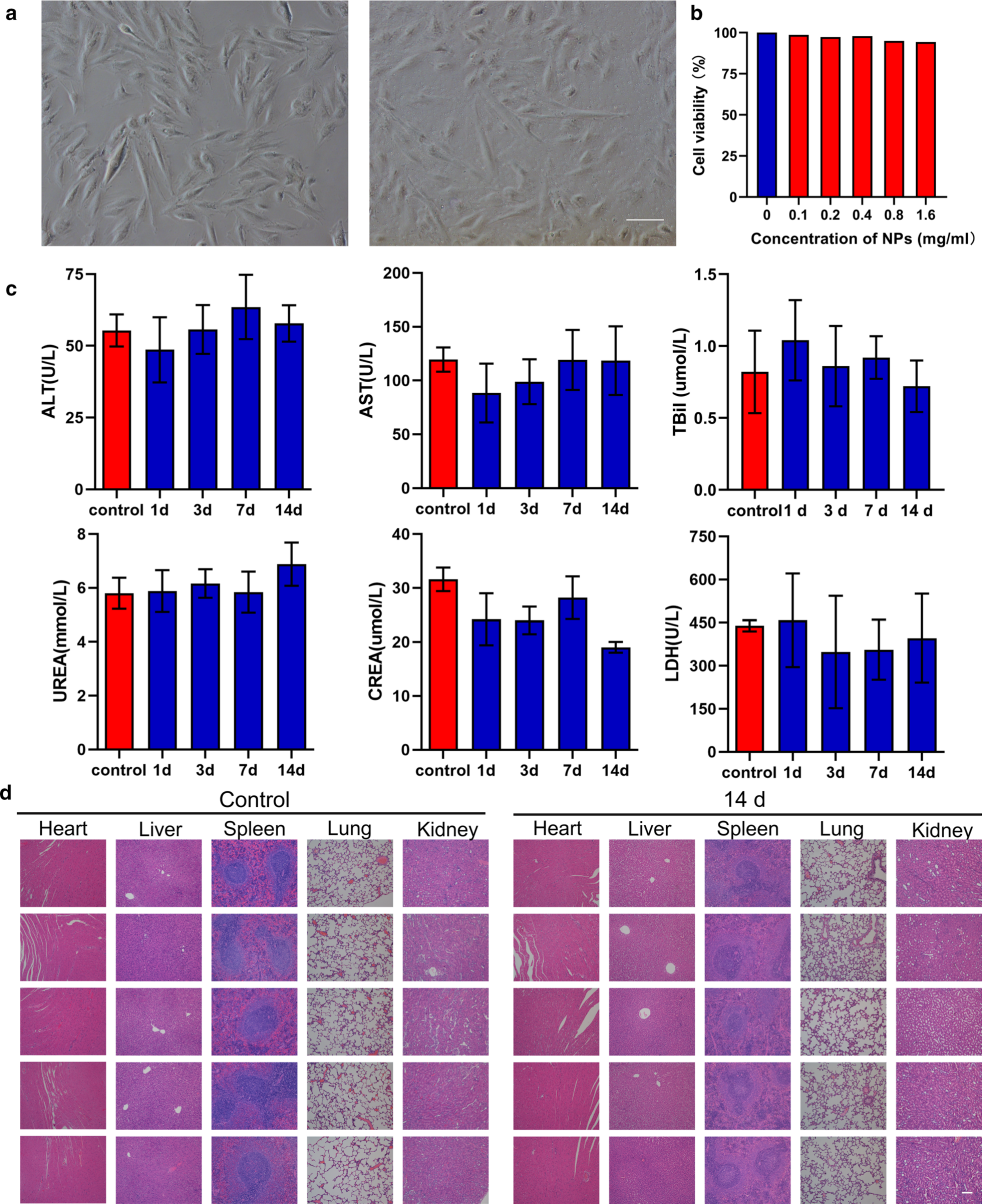
The safety profile of NPs. **a** Morphology of normal H9C2 cells and co-incubation with IMTP-Fe_3_O_4_-PFH NPs (scale bar: 100 μm). **b** Cell viability of H9C2 cells after co-incubation with different concentrations of IMTP-Fe_3_O_4_-PFH NPs. **c** Blood biochemical indexes analysis of SD rats from the control group and the experimental groups at 1, 3, 7, and 14 d post intravenous injection of IMTP-Fe_3_O_4_-PFH NPs. **d** HE staining of major organs from the control group and 14 d experimental group after 14 days of intravenous administration with IMTP-Fe_3_O_4_-PFH NPs (scale bar: 100 μm)

Considering the subsequent use of NPs both in vitro and in vivo, the safety evaluation of IMTP-Fe_3_O_4_-PFH NPs was important. The cytotoxicity of NPs was confirmed in H9C2 cell incubated by light microscopy and CCK-8 assay. No change in cell morphology and viability compared to the group without NPs, even at the maximum concentration **(**Fig. 2a, b). The in vivo biosafety was further assessed in rats after intravenous administration of IMTP-Fe_3_O_4_-PFH NPs. Throughout this observation period, no abnormal behavior or mortality was observed in all rats. At different time points, there were negligible differences among the different groups in various blood indexes, including functional markers of the liver (ALT, AST, TB), kidney (BUN, CR), myocardial enzyme spectrum (LDH) and blood routine indexes (RBC, WBC, PLT, HGB, MCV) (Fig. 2c, Additional file 1: Figure S3). Besides, after 14 days, HE staining of important organs such as the heart, liver, spleen, lungs, and kidneys showed no change compared to the normal group (Fig. 2d). The above results all confirmed the good biocompatibility of NPs, no obvious toxicity compared with the control group, thus ensuring safety in subsequent experiments.

### Targeting efficiency and bio-distribution of the NPs

The NPs could be accumulated in the ischemic myocardium because of the ischemia-induced enhanced permeability and retention (EPR) effect, which was the main mechanism for passively targeting the ischemic region of the myocardium [38, 46, 47]. Besides, targeting a specific location could be used to improve payload delivery and residence time, which was a vital step to achieve the desired effects in particular [48]. The NPs combination with IMTP played such a role.

**Fig. 3:**
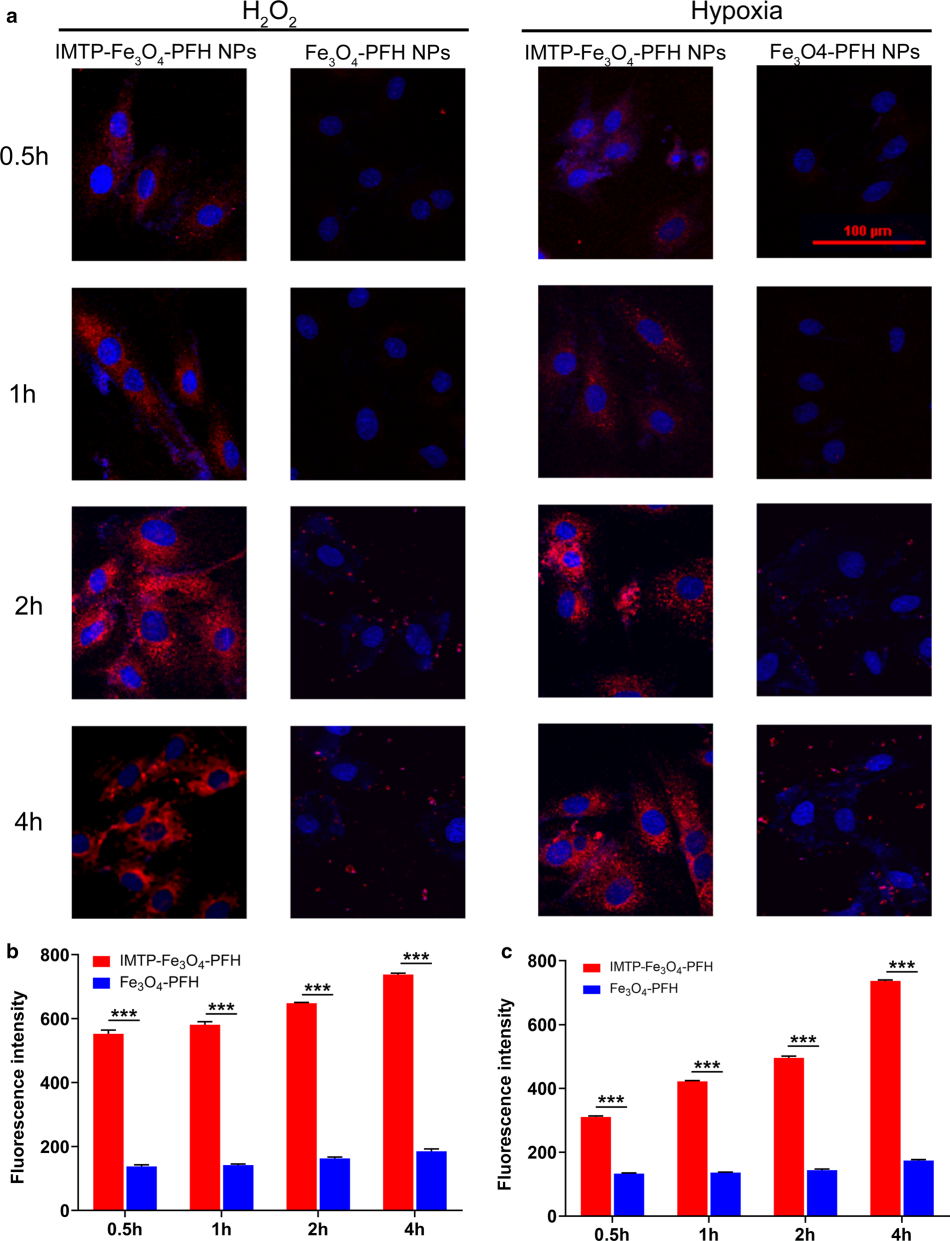
In vitro targeting efficiency. **a** CLSM images of H9C2 cells and IMTP-Fe_3_O_4_-PFH NPs or Fe_3_O_4_-PFH NPs incubated at different time points after cells subjected to hypoxia or H_2_O_2_ (scale bar: 100 μm). **b** Fluorescence intensity of H9C2 cells subjected to H_2_O_2_ incubated with IMTP-Fe_3_O_4_-PFH NPs or Fe_3_O_4_-PFH NPs for different durations (***p < 0.001). **c** Fluorescence intensity of H9C2 cells subjected to hypoxia incubated with IMTP-Fe_3_O_4_-PFH NPs or Fe_3_O_4_-PFH NPs for different durations

Considering that the mechanism of IMTP was unknown, both hypoxia and H_2_O_2_ were chosen for modeling. According to the microscopic cell morphology and CCK-8 detection of cell viability, hypoxia for 24 h and 5 µmol/l of H_2_O_2_ were finally chosen as the modeling conditions (Additional file 1: Figure S4, S5). Building on the success of cell modeling as expected, the CLSM images showed that IMTP-Fe_3_O_4_-PFH NPs efficiently surrounded the H9C2 cells and became more pronounced with time, while Fe_3_O_4_-PFH NPs were scattered and disordered (Fig. 3a). The fluorescence intensity measured also demonstrated that after 4 h of incubation, the intensity of targeted NPs was maximized in both the H_2_O_2_ group and hypoxic group, and was statistically significant compared to the non-targeted groups (Fig. 3b, c).

**Fig. 4:**
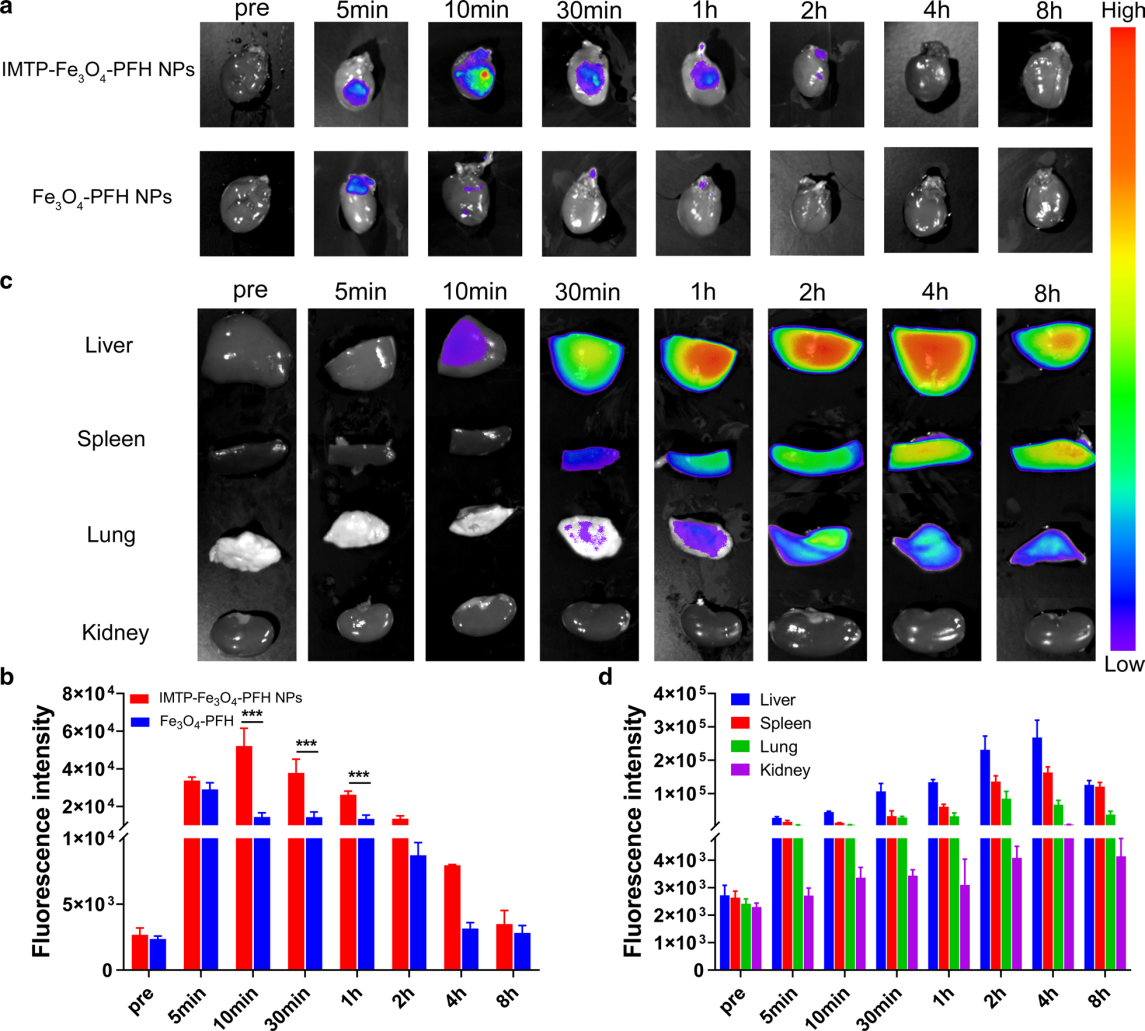
Ex vivo targeting ability and biodistribution. Ex vivo fluorescence images (**a**) and quantitative analysis (**b**) of the hearts of model rats at different time points after intravenous administration of IMTP-Fe_3_O_4_-PFH NPs or Fe_3_O_4_-PFH NPs (***p < 0.001). **c** Ex vivo fluorescence images of the distribution of IMTP-Fe_3_O_4_-PFH NPs in the main organs (liver, spleen, lung, and kidney) at different time points. **d** Quantitative biodistribution analysis of IMTP-Fe_3_O_4_-PFH NPs in the main organs

The IMTP-Fe_3_O_4_-PFH NPs specificity of targeted and bio-distribution were also validated by the ex vivo fluorescence experiment. In preliminary exploration, the model of myocardial ischemia in rats had been verified to be successful and repeatable through the intraoperative and postoperative index (Additional file 1: Figure S6, Additional file 2: Movie S1 and Movie S2). After injection of DiR-labeled IMTP-Fe_3_O_4_-PFH NPs, fluorescent detection showed that signal at the damaged heart site gradually increased starting at 5 min, peaking at 10 min, and then decreasing at 30 min until it disappeared completely after 4 h (Fig. 4a). Conversely, no significant fluorescence was seen in the non-targeted group to accumulate in the anterior wall of the left ventricle at all detection time points. Quantitative analysis of the fluorescence intensity demonstrated once again a huge difference (*P* < 0.001) between the two groups from 10 min to 1 h (Fig. 4b). These data indicated that IMTP conjugation was able to increase the on-target delivery of systemically administered NPs. Moreover, based on the occurrence of max fluorescence intensity, it provided a basis for choosing 10 min post-injection as the appropriate time point for subsequent multimodal imaging.

As shown in Fig. 4c, the IMTP-Fe_3_O_4_-PFH NPs gradually distributed to most of the organs from 30 min onwards, mainly to the liver, spleen, and lungs. The quantitative analysis further confirmed the results (Fig. 4d). The liver as the major metabolic organ, the fluorescence intensity of it peaked at 4 h and then gradually decreased.

### ADV and US imaging in vitro and in vivo

**Fig. 5:**
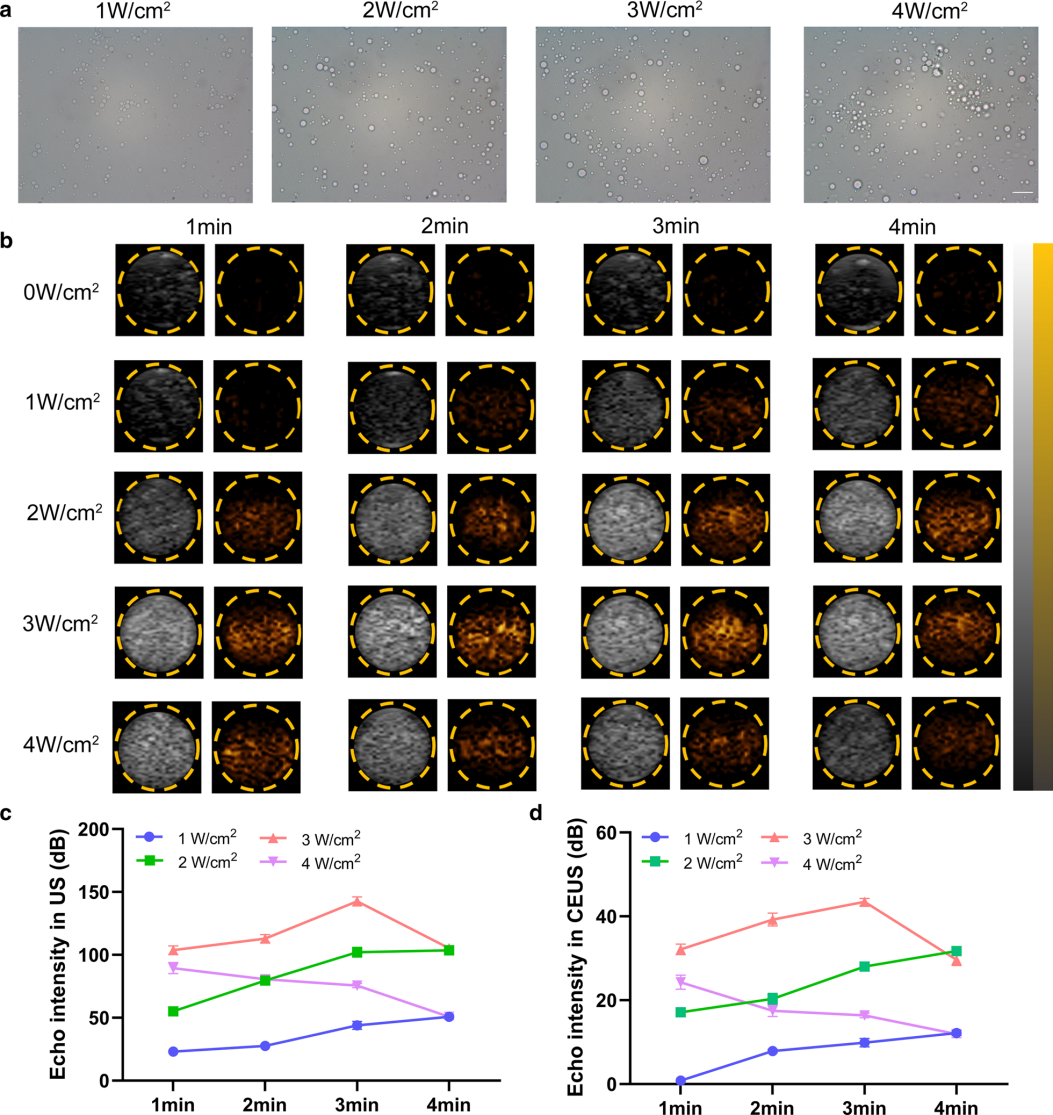
Phase transition and US imaging in vitro. **a** Light microscope images of phase transformation induced by LIFU (scale bar = 10 μm). **b** The US imaging (left: B-Mode, right: CEUS) of IMTP-Fe_3_O_4_-PFH NPs with time- and intensity-dependent ADV. Quantitative analysis of the echo intensity of NPs after LIFU irradiation at different intensities and time in B-mode (**c**) and CEUS-mode (**d**)

PFH has been demonstrated to be an ideal phase-transformation material, which can be triggered by many factors (US, temperature, lasers), but it retains its gaseous state at both room and body temperature with a boiling point of 56 ℃ [49, 50]. In IMTP-Fe_3_O_4_-PFH NPs, PFH played a role due to ADV triggered by LIFU, thereby allowing PFH-coated NPs to enhance US imaging. Firstly, the NPs visualized with optical microscopy detected no obvious change at the intensity of 1 W/cm^2^. However, with increasing intensity, the NPs increased in size until they broke up at 4 W/cm^2^ (Fig. 5a). The in vitro US imaging performance of IMTP-Fe_3_O_4_-PFH NPs was analyzed, showing a similar trend as above (Fig. 5b). Before LIFU irradiation, both in B-mode and CEUS-mode there showed no echo or very low echo intensity. Starting at a density of 2 W/cm^2^, the echo intensity of NPs increased with time and reached its peak at 3 W/cm^2^. After LIFU irradiation at 4 W/cm^2^, however, the intensity decreased, which presumably due to the rupture of some NPs when their volume grew to a certain size. The corresponding echogenic intensity was quantized by analytical software and the results were consistent with their in vitro performances (Fig. 5c, d). In the meanwhile, the echo intensity of NPs without PFH did not change, regardless of LIFU intensity or irritation time both in B-mode and CEUS mode (Additional file 1: Figure S7). The above results all implied that IMTP-Fe_3_O_4_-PFH NPs encapsulated PFH could have a good response to ADV, and subsequently enhanced US imaging performances.

**Fig. 6:**
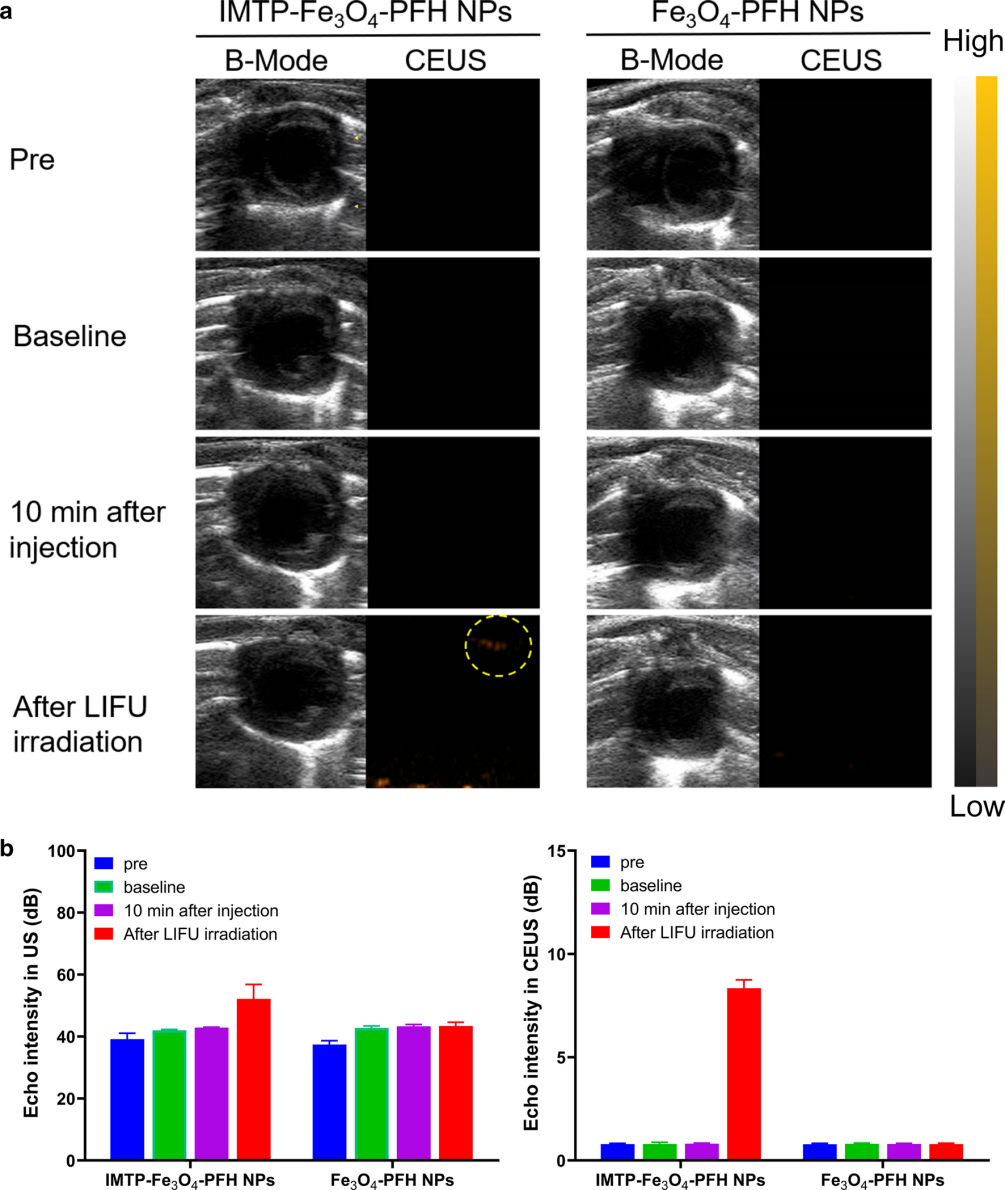
In vivo US imaging. **a** B-Mode and CEUS images of hearts in model rat pre-operation, baseline, 10 min after injection, and after LIFU irradiation. **b** Corresponding echo intensity in B-Mode (left) and CEUS mode (right)

In acute myocardial ischemia, the abnormal segmental motion of the ventricular wall may be seen in B-mode. However, in some cases, reduced motion in the lesion area is not atypical, thus limiting the use of US imaging in the diagnosis of acute myocardial ischemia. Therefore, the application of molecular probes may be a new exploration. Referring to the preceding bio-distribution of NPs in vivo, the time point of 10 min post-injection NPs was chosen for observation, and no obvious US enhancement was observed in either IMTP-Fe_3_O_4_-PFH NPs or Fe_3_O_4_-PFH NPs group (Fig. 6a). After LIFU irradiation to the heart with the intensity of 3 W/cm^2^ for 3 min, a bright signal occurred at the diseased site under CEUS mode in the IMTP-Fe_3_O_4_-PFH NPs group but not in the Fe_3_O_4_-PFH NPs group. The corresponding echo intensity in the targeted group was substantially higher than that in the non-targeted group (Fig. 6b). These results were consistent with previous studies [51], and indicated that without LIFU irritation, NPs could not initiate ADV; as the same, without IMTP for targeting, nor could they enhance the US imaging at the ischemic myocardium.

### In vitro and in vivo PA imaging

**Fig. 7:**
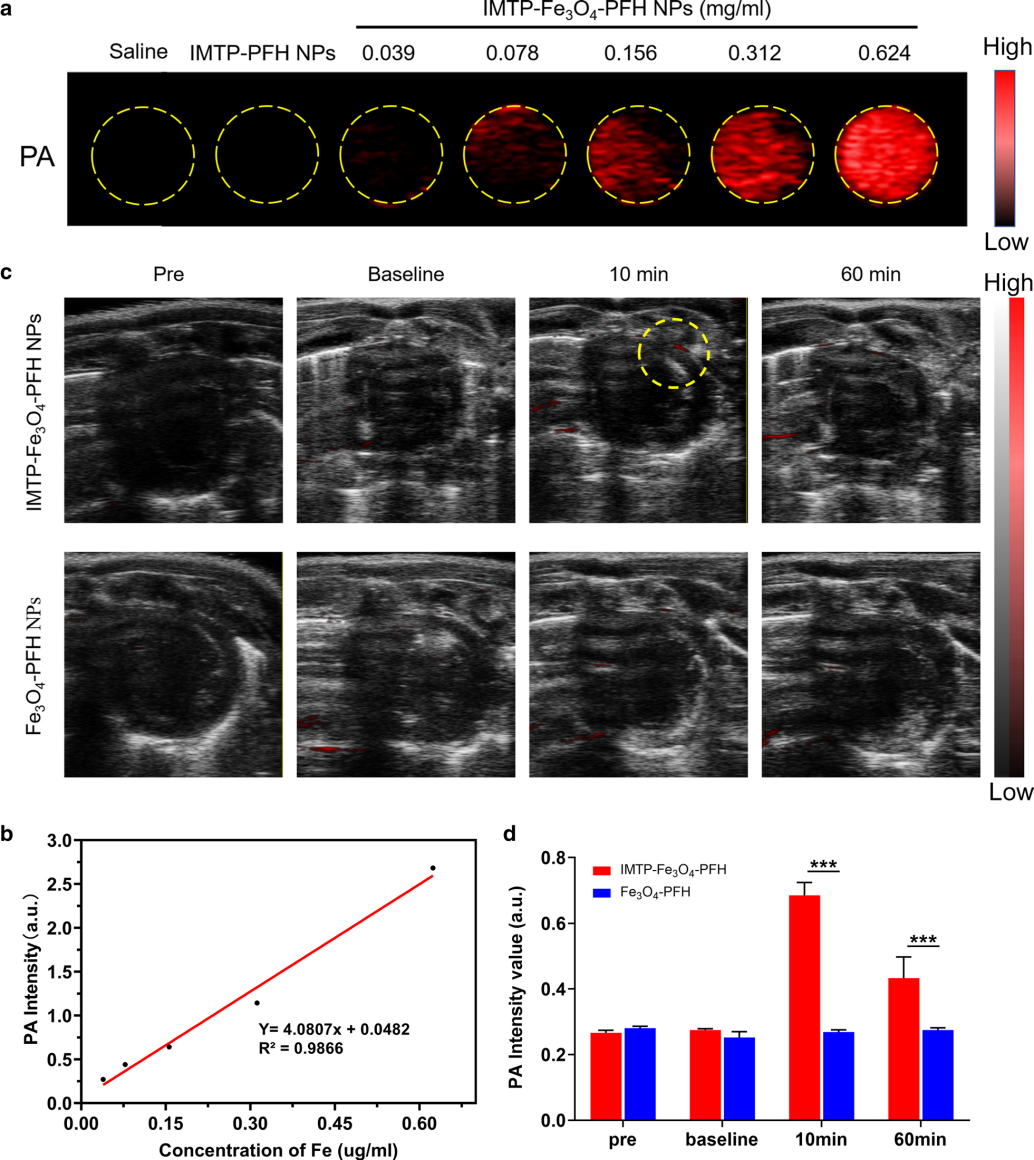
PA imaging in vitro and in vivo. PA images (**a**) and PA values (**b**) of IMTP-Fe_3_O_4_-PFH NPs at different Fe concentrations in vitro. **c** In vivo PA images of the hearts in model rats after intravenous injection of IMTP-Fe_3_O_4_-PFH NPs or Fe_3_O_4_-PFH NPs pre-operation, baseline, 10 min, and 60 min after injection. **d** The changes of PA signal intensity in the heart region at the corresponding time point

To select maximum absorbance, the IMTP-Fe_3_O_4_-PFH NPs were first scanned at full spectrum ranging from 680 to 950 nm. The peak appeared at 690 nm, which was close to previously reported [32], so this wavelength was selected for subsequent PA imaging (Additional file 1: Figure S8). As displayed in Fig. 7a, it was found that IMTP-Fe_3_O_4_-PFH NPs showed the concentration-dependent contrast enhancement in PA imaging while the NPs without Fe_3_O_4_ represented no signal, indicating that the presence of Fe_3_O_4_ was critical for the PA performance. Besides, in the quantitative analysis of the PA signal, the intensity was linear with increasing Fe concentration (Fig. 7b).

In vivo, PA imaging was performed on the heart in ischemic rat models before and after intravenous injection IMTP-Fe_3_O_4_-PFH NPs or Fe_3_O_4_-PFH NPs (Fig. 7c), and the PA value was quantitatively analyzed (Fig. 7d). The PA signal at the ischemic myocardium was visible only in IMTP-Fe_3_O_4_-PFH NPs group, indicating the effective accumulation of targeted NPs within the ischemic region. Based on these results, IMTP-Fe_3_O_4_-PFH NPs could be used for imaging-guided therapy in ischemic cardiomyopathy under the PA imaging.

### In vitro and in vivo MR imaging performance of IMTP-Fe_3_O_4_-PFH NPs

**Fig. 8:**
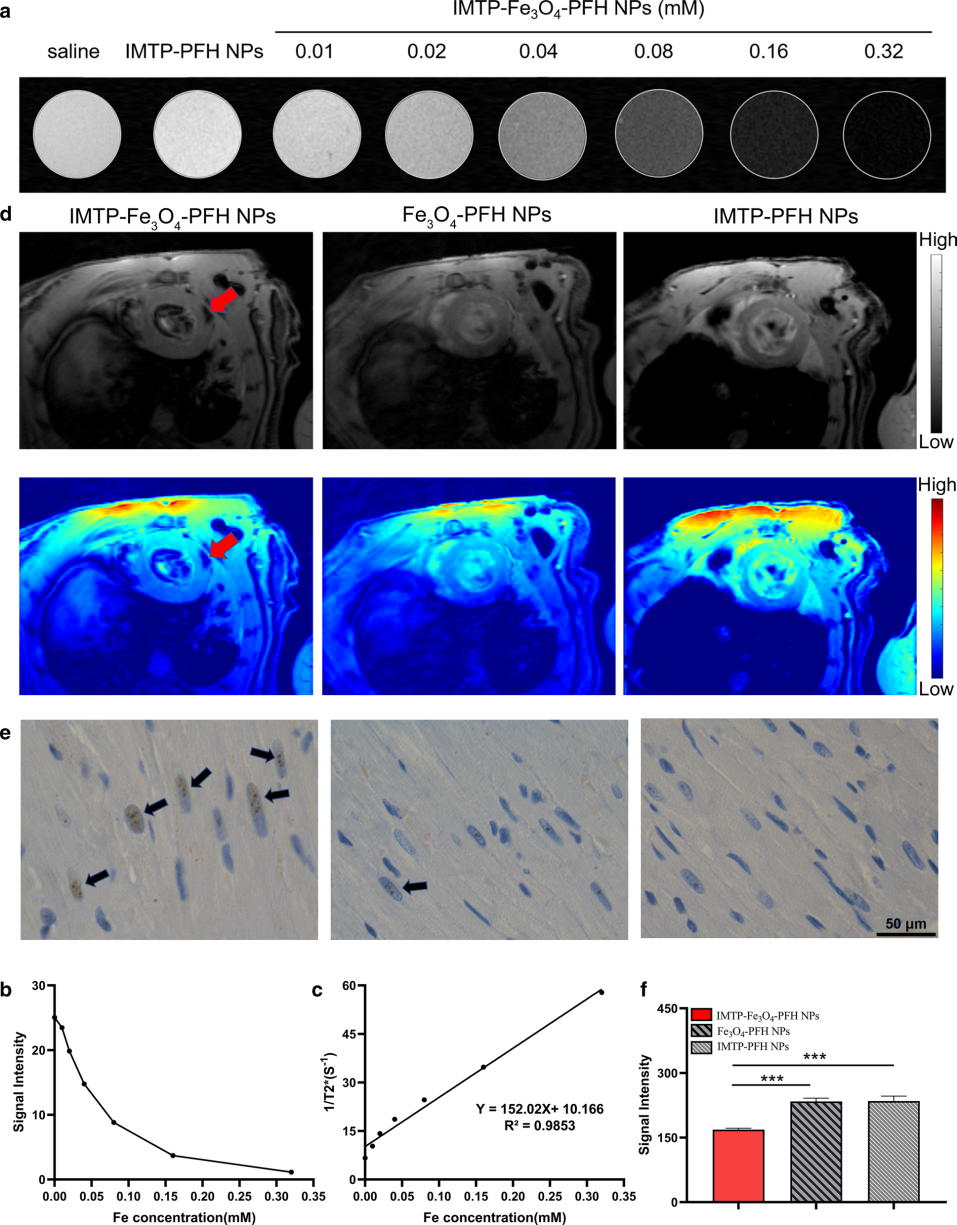
MR imaging in vitro and in vivo. T_2_-WI (**a**) and signal intensity (**b**) of IMTP-Fe_3_O_4_-PFH NPs at different Fe concentrations in vitro. **c** The curve of 1/T_2_ versus Fe concentration under T_2_-weighted MR scanning. The slope indicated the specific relativity r_2_. **d** T_2_*WI of the hearts after intravenous injection of IMTP-Fe_3_O_4_-PFH NPs, Fe_3_O_4_-PFH NPs, and IMTP -PFH NPs respectively. The top row showed grayscale images, and the bottom row showed the pseudo-coloring images. Red arrows pointed to the ischemic myocardium area. **e** Prussian blue & DAB staining of the ischemic myocardium area in different groups after MR imaging (scale bar = 50 μm). Black arrows pointed to brown stained iron. **f** Quantitative analysis of enhanced cardiac MR signal intensity between each group in vivo

SPIONs as negative contrast agents for T_2_-WI are clearly effective due to the strong signal and low endogenous background [29]. As expected, the IMTP-Fe_3_O_4_-PFH NPs at different concentrations (0.01, 0.02, 0.04, 0.08, 0.16, and 0.32 mM), even at very low concentrations, could show in great negative enhancements in T_2_-WI in vitro (Fig. 8a), and the signal intensity gradually diminished with increasing concentration (Fig. 8b). This change was not observed in saline and NPs without Fe_3_O_4_. The transverse relativity r_2_ was calculated to be 152.02 mM^−1^·s^−1^ (Fig. 8c), which was higher than previously commercially MRI contrast agents, such as Feridex (98.3 mM^−1^·s^−1^).

After surgery in rats, NPs were injected into each group via tail vein, followed by MR imaging to measure the ability to image and diagnose the infarcted region of the myocardium. The Fe_3_O_4_-PFH NPs or IMTP-PFH NPs group didn’t show any obvious change of signal intensity. However, the notable negative signal enhancement could be seen in the infarcted area after being treated with IMTP-Fe_3_O_4_-PFH NPs, and pseudo-coloring of the grayscale image was performed to further clearly show this phenomenon, which indicated that IMTP-Fe_3_O_4_-PFH NPs could target the infarcted area (Fig. 8d). Quantifying MR imaging induced by NPs accumulation in the injured region was got by assessing the relative signal intensity (Fig. 8f). To validate the accumulation, cross-sections from the imaging hearts were stained with Prussian blue & DAB. As shown in Fig. 8e, the brown staining (black arrows) mainly observed in the ischemic myocardium with IMTP-Fe_3_O_4_-PFH NPs**,** while little blue staining was exhibited in the Fe_3_O_4_-PFH NPs group and no staining showed in the IMTP-PFH NPs group.

## Conclusion

Multimodal imaging uses two or more imaging modalities into one system to obtain more comprehensive and accurate information in clinical diagnostic imaging by integrating optical, acoustic, nuclear, and magnetic technologies [52]. For example, PET-CT and PET-MRI imaging have been successfully used in clinical. US imaging as an irreplaceable tool in the diagnosis of cardiovascular diseases and PA imaging, a top hit of optical imaging in recent years, have strong complementary properties. PA imaging has advantage of the high sensitivity and contrast, while US imaging compensates for its limited depth of detection [53]. Besides, MR imaging can provide high resolution. Our study is the first to use these three imaging modalities to detect ischemic myocardium, enabling complementary advantages among them. IMTP-Fe_3_O_4_-PFH NPs we have successfully synthesized as a new non-invasive probe not only has a high safety profile in vitro and in vivo, but is indeed capable of target on H9C2 cells both in hypoxia and H_2_O_2_ for modeling. Similarly, there was a clear significant targetability in rats for in vivo hearts. Based on the unique ADV effect of PFH, these nanodroplets developed a gas–liquid phase transition under LIFU irradiation, altering the acoustic environment to enhance US imaging. Furthermore, the special properties of Fe_3_O_4_ acted as a sonosensitizer in PA imaging and a contrast agent in MR imaging. All the findings in this work indicated that IMTP-Fe_3_O_4_-PFH NPs could be a promising multifunctional probe, thus achieving an accurate diagnosis of ischemic myocardium and laying the foundation for future stem cell tracing, targeting gene transfection and precise drug delivery in vivo.

## Supplementary Information

**Additional file 1**: **Figure S1**. The size changes of IMTP-Fe_3_O_4_-PFH NPs for different days. **Figure S2**. The iron standard curves by atomic absorption spectroscopy. **Figure S3**. The routine blood indexes in the control group and the experimental groups. **Figure S4**. Morphology of normal H9C2 cells and cells treated with hypoxia for 24 h and 5 µmol/l of H_2_O_2_. **Figure S5**. Cell viability of H9C2 cells after treated with hypoxia for different duration and co-incubation with different concentrations of H_2_O_2_. **Figure S6**. Intra-operation and post-operation verification of ischemic myocardial model in rats. **Figure S7**. ADV and US imaging of IMTP-Fe_3_O_4_ NPs at different intensities of LIFU irritation and different time in vitro. **Figure S8**. The PA signal changes of IMTP-Fe_3_O_4_-PFH NPs irradiated by a laser at full spectrum ranging from 680 to 950 nm.

**Additional file 2**: **Movie S1**. Echocardiography before operation in rats. **Movie S2**. Echocardiography after operation in model rats.

## Data Availability

All data generated or analyzed during this study are included in this published article and its additional file 1 and 2.
