# Correction: A non‑invasive nanoparticles for multimodal imaging of ischemic myocardium in rats

**DOI:** 10.1186/s12951-022-01588-2

**Published:** 2022-08-23

**Authors:** Xiajing Chen, Yanan Zhang, Hui Zhang, Liang Zhang, Lingjuan Liu, Yang Cao, Haitao Ran, Jie Tian

**Affiliations:** 1 Department of Cardiology, Ministry of Education Key Laboratory of Child Development and Disorders, National Clinical Research Center for Child Health and Disorders (Chongqing), China International Science and Technology Cooperation Base of Child Development and Critical Disorders, Children’s Hospital of Chongqing Medical University, Chongqing, 400014 People’s Republic of China; 2 Chongqing Key Laboratory of Pediatrics, Children’s Hospital of Chongqing Medical University, Chongqing, 400014 People’s Republic of China; 3 Chongqing Key Laboratory of Ultrasound Molecular Imaging & Department of Ultrasound, The Second Affiliated Hospital of Chongqing Medical University, Chongqing, 400010 People’s Republic of China

## Correction: J Nanobiotechnol (2021) 19:82 10.1186/s12951-021-00822-7

After the publication of the original article [1], the authors have identified mistakes in Fig. 2d, Fig. 5b, and Additional file 1: Figure S7. The revised figures are shown in this correction. All authors sincerely apologize for these errors.

**Fig. 2:**
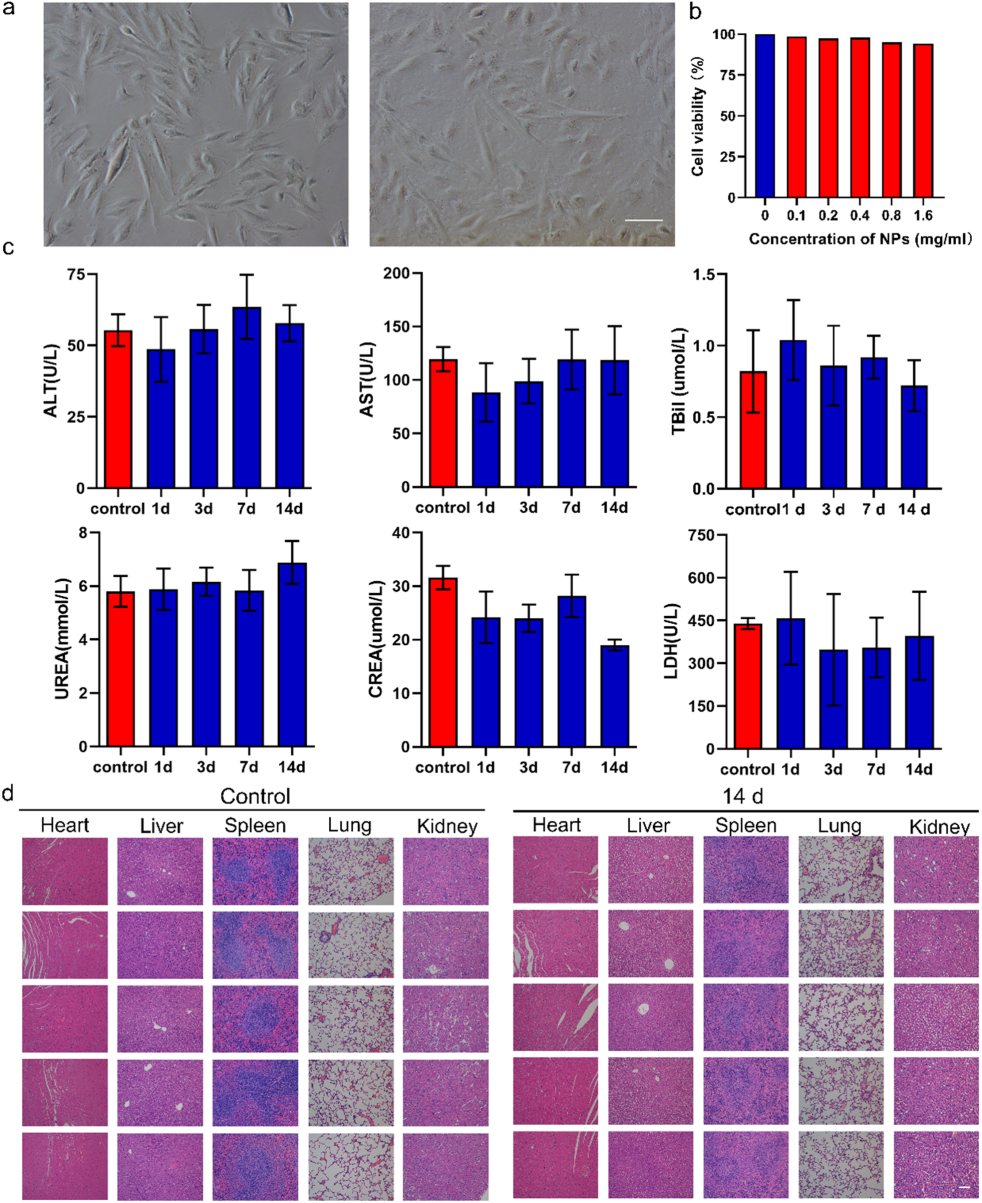
The safety profile of NPs. **a** Morphology of normal H9C2 cells and co-incubation with IMTP-Fe_3_O_4_-PFH NPs (scale bar: 100 μm). **b** Cell viability of H9C2 cells after co-incubation with different concentrations of IMTP-Fe_3_O_4_-PFH NPs. **c** Blood biochemical indexes analysis of SD rats from the control group and the experimental groups at 1, 3, 7, and 14 d post intravenous injection of IMTP-Fe_3_O_4_-PFH NPs. **d** HE staining of major organs from the control group and 14 d experimental group after 14 d of intravenous administration with IMTP-Fe_3_O_4_-PFH NPs (scale bar: 100 μm)

**Fig. 5:**
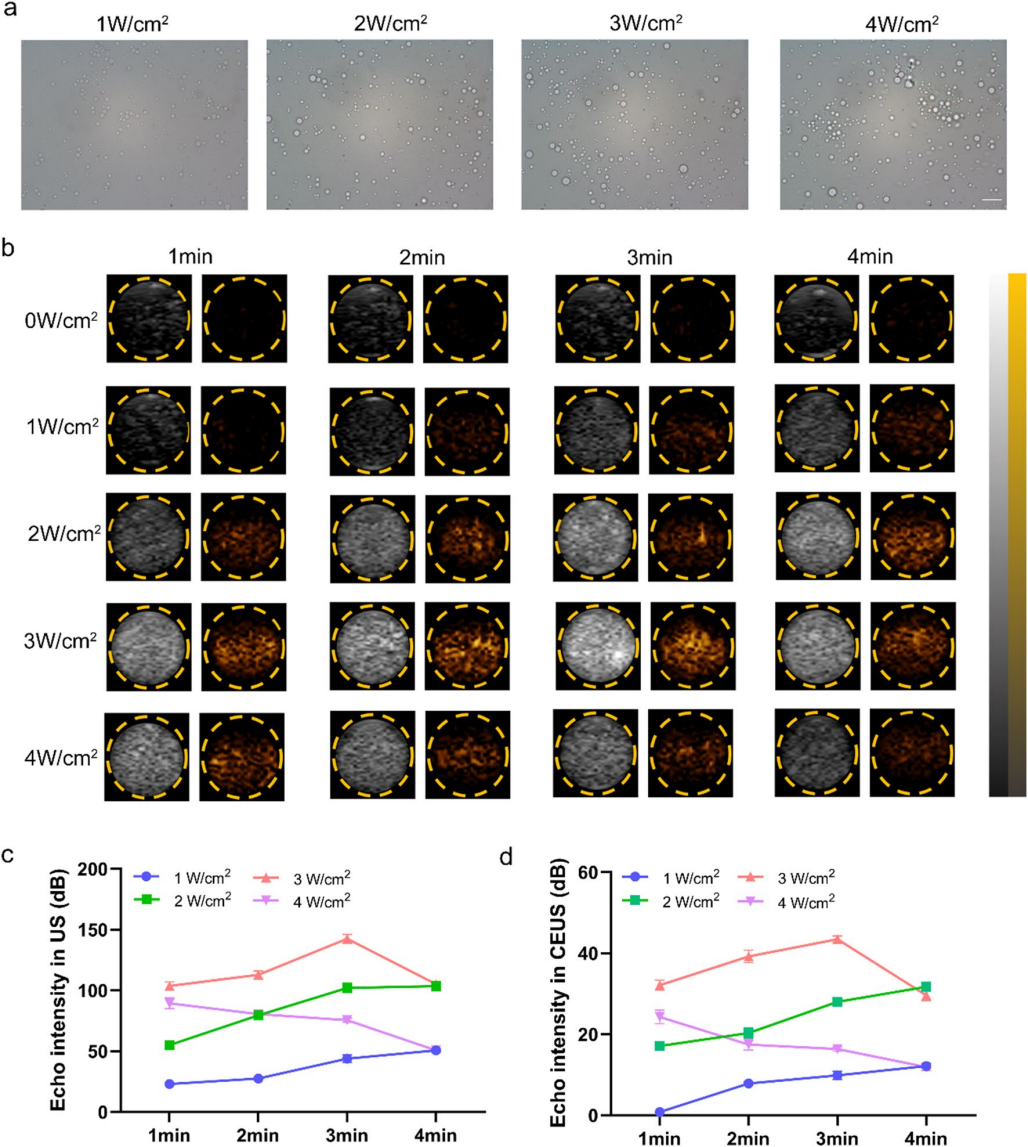
Phase transition and US imaging in vitro. **a** Light microscope images of phase transformation induced by LIFU (scale bar = 10 μm). **b** The ultrasound imaging (left: B-Mode, right: CEUS) of IMTP-Fe_3_O_4_-PFH NPs with time- and intensity-dependent ADV. Quantitative analysis of the echo intensity of NPs after LIFU irradiation at different intensities and time in B-mode (**c**) and CEUS-mode (**d**)

## Supplementary Information

**Additional file 1: Figure S7.** ADV and US imaging of IMTP-Fe_3_O_4_ NPs at different intensities of LIFU irritation and different time in vitro. Echo intensity did not change in all cases.
