# Supplementary material for: A non-invasive nanoparticles for multimodal imaging of ischemic myocardium in rats
Source: J Nanobiotechnology. 2021 Mar 22;19:82. doi: 10.1186/s12951-021-00822-7 (PMC7986298; doi:10.1186/s12951-021-00822-7)
Supplement: Supplementary file 1 — Additional file 1: Figure S1. The size changes of IMTP-Fe3O4-PFH NPs for different days. Figure S2. The iron standard curves by atomic absorption spectroscopy. Figure S3. The routine blood indexes in the control group and the experimental groups. Figure S4. Morphology of normal H9C2 cells and cells treated with hypoxia for 24 h and 5 µmol/l of H2O2. Figure S5. Cell viability of H9C2 cells after treated with hypoxia for different duration and co-incubation with different concentrations of H2O2. Figure S6. Intra-operation and post-operation verification of ischemic myocardial model in rats. Figure S7. ADV and US imaging of IMTP-Fe3O4 NPs at different intensities of LIFU irritation and different time in vitro. Figure S8. The PA signal changes of IMTP-Fe3O4-PFH NPs irradiated by a laser at full spectrum ranging from 680 to 950 nm. [file 12951_2021_822_MOESM1_ESM.docx]

**Additional file 1**

# A Non-Invasive Nanoparticles For Multimodal Imaging Of Ischemic Myocardium In Rats

Xiajing Chen^1,2^, Yanan Zhang^1,2^, Hui Zhang^1,2^, Liang Zhang^3^, Lingjuan Liu^1,2^, Yang Cao^3^, Haitao Ran^3^, Jie Tian^1,2,*^

^1^Department of Cardiology; Ministry of Education Key Laboratory of Child Development and Disorders; National Clinical Research Center for Child Health and Disorders (Chongqing); China International Science and Technology Cooperation base of Child development and Critical Disorders; Children’s Hospital of Chongqing Medical University; Chongqing 400014, People’s Republic of China;

^2^Chongqing Key Laboratory of Pediatrics, Children’s Hospital of Chongqing Medical University, Chongqing,400014, People’s Republic of China;

^3^Chongqing Key Laboratory of Ultrasound Molecular Imaging & Department of Ultrasound, the Second Affiliated Hospital of Chongqing Medical University, Chongqing 400010, People’s Republic of China

*Corresponding Author: Jie Tian. Email: [jietian@cqmu.edu.cn](mailto:jietian@cqmu.edu.cn)

**
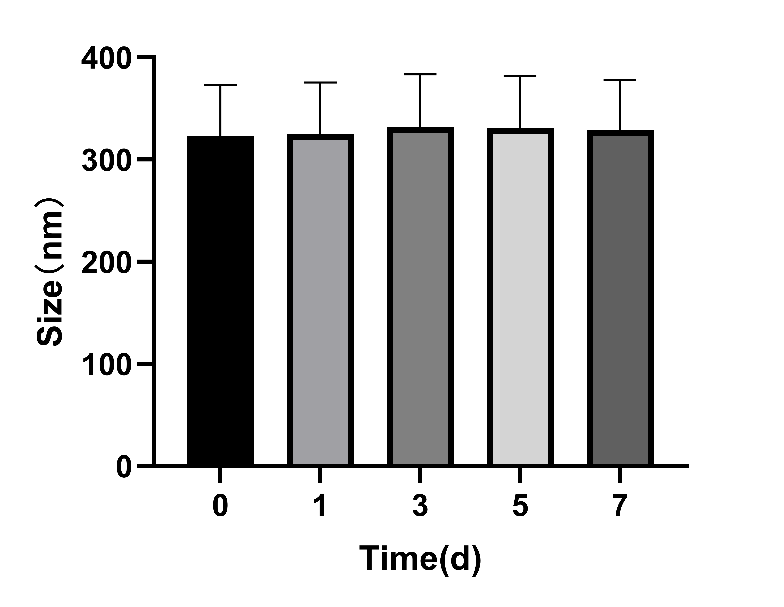
****Figure S1**. The size changes of IMTP-Fe_3_O_4_-PFH NPs for different days.

**
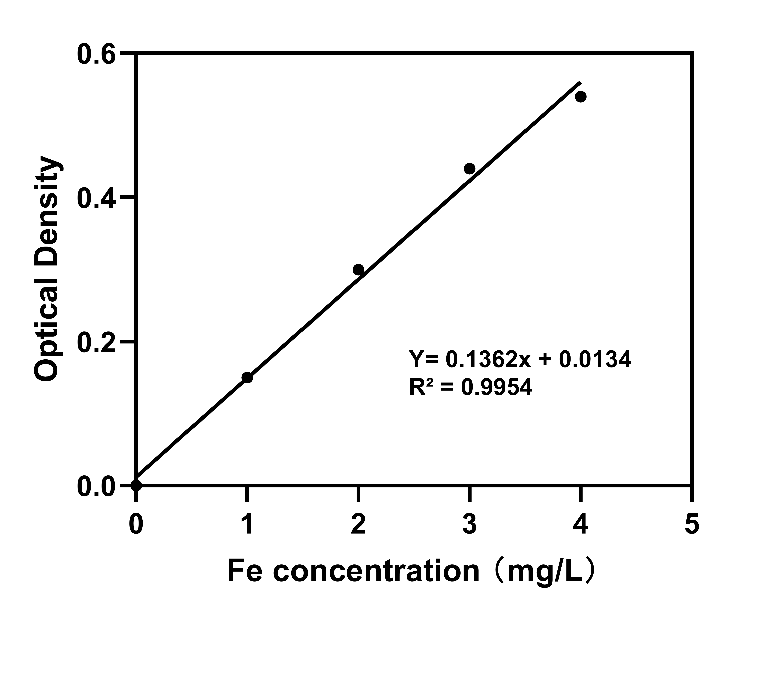
****Figure S2.** The iron standard curves by atomic absorption spectroscopy.

**
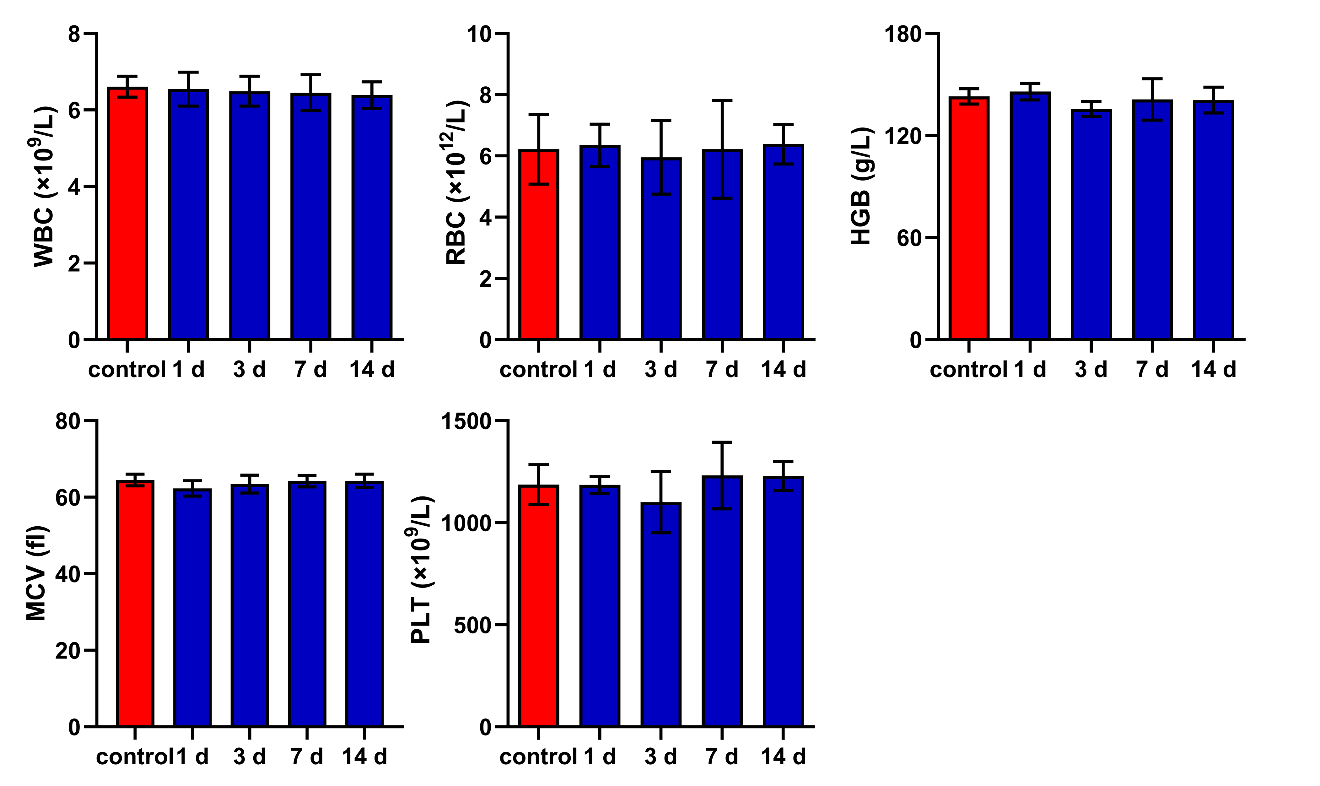
Figure S3.** The routine blood indexes in the control group (1 d, intravenous injection saline) and the experimental groups (1, 3, 7, 14 d, intravenous injection of IMTP-Fe_3_O_4_-PFH NPs) (n = 5). At different time points, there was no difference in each blood routine index among groups.

**
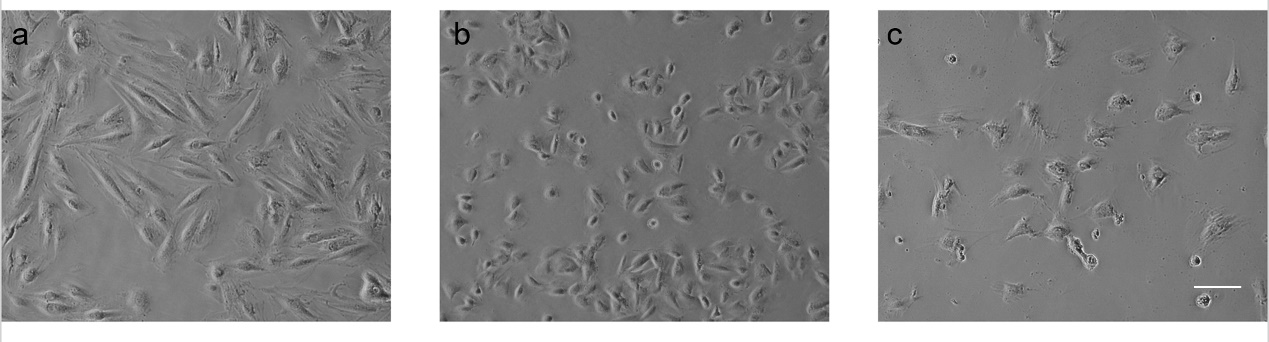
****Figure S4.** Morphology of normal H9C2 cells (a) and cells treated with hypoxia for 24 h (b) and 5 µmol/l of H_2_O_2_ (c) (scale bar: 100 μm). Compared with the normal group, H9C2 cells in both treated groups showed wrinkling and shedding, and the number of cells was significantly reduced, thus proving the success of cell modeling.

**
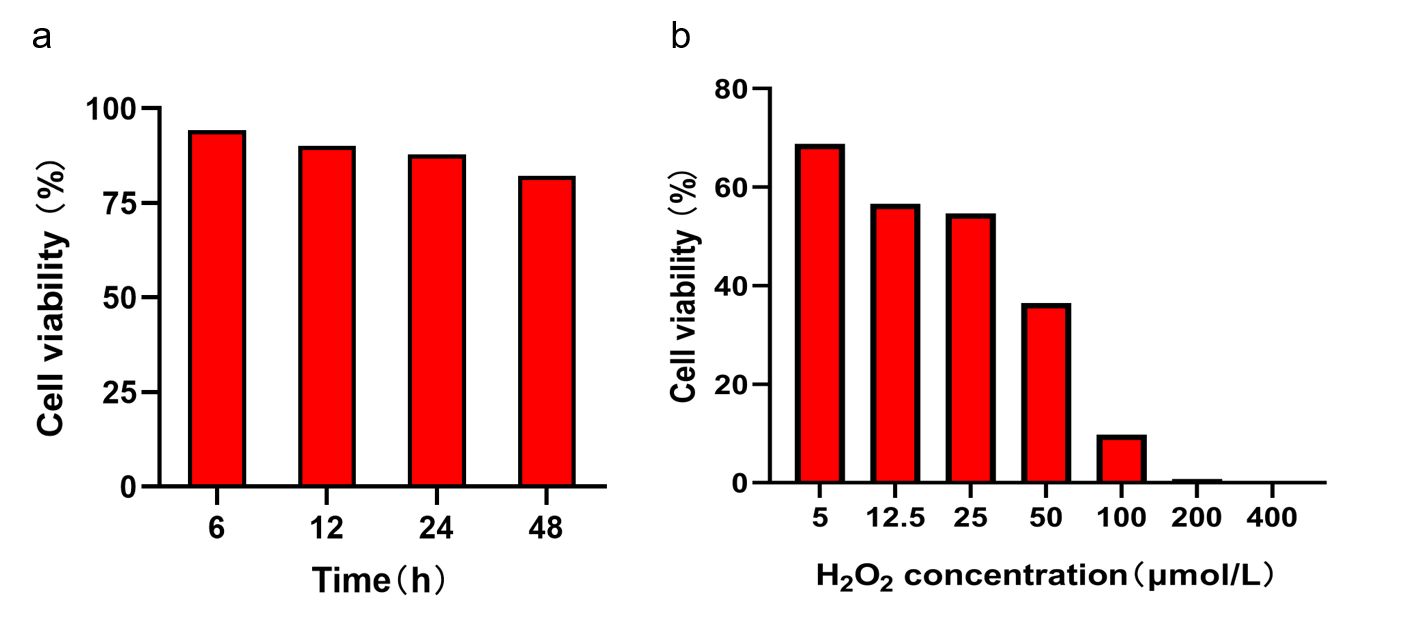
Figure S5.** Cell viability of H9C2 cells after treated with hypoxia for different duration (a) and co-incubation with different concentrations of H_2_O_2_ (b). In the hypoxic injury model, cell viability gradually decreased with increasing hypoxic time, especially from 24 hours onwards, damage was obvious. In the H_2_O_2_-induced cell injury model, the effect of H_2_O_2_ on cells was seen to be significant, with almost all cells dying at high concentrations and cell viability significantly decreased to 68.78% even at the lowest concentration (5 μmol/L).

**
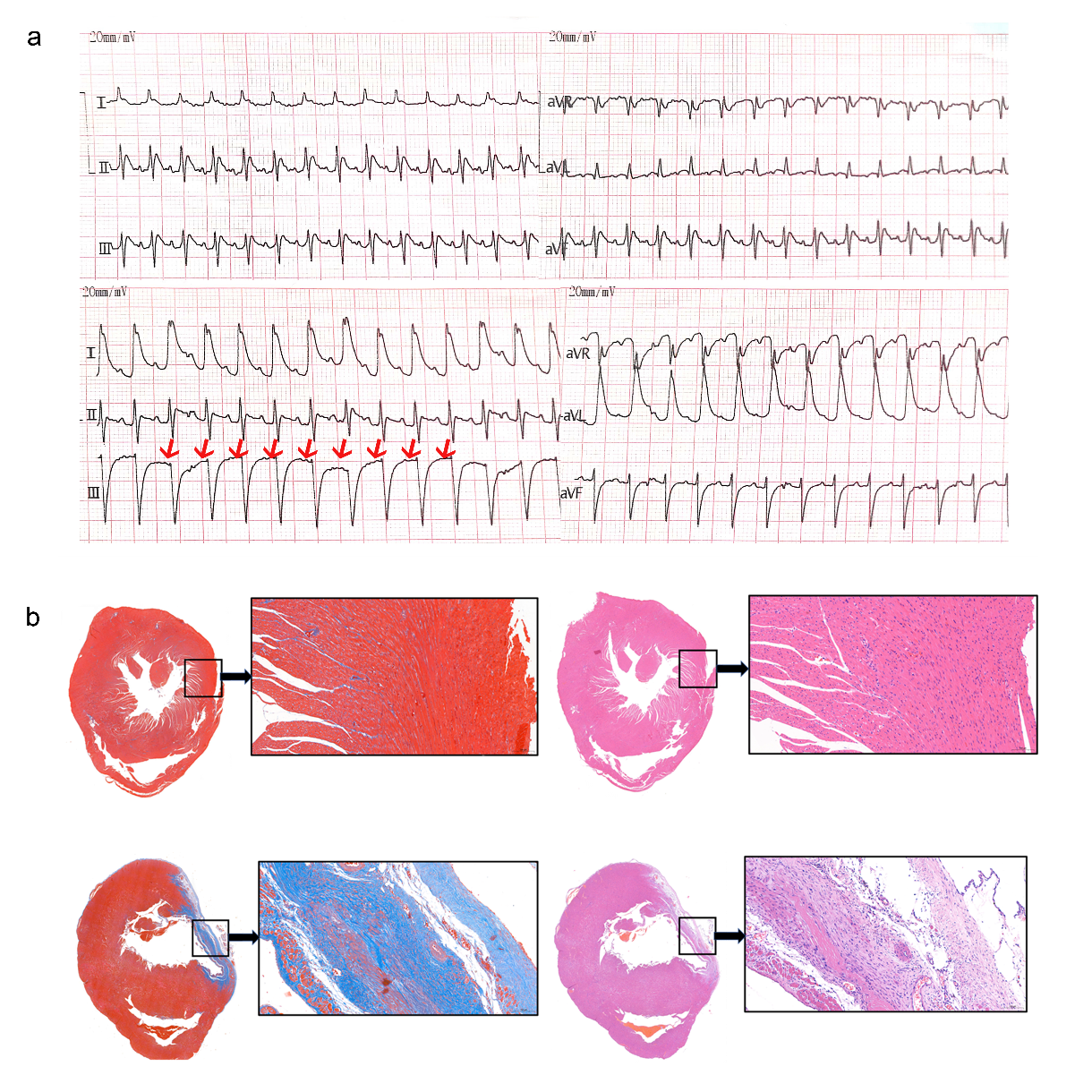
Figure S6.** Intra-operation and post-operation verification of ischemic myocardial model in rats. (a) Changes of electrocardiogram before and after coronary ligation (top row: pre- ligation, bottom row: post-ligation). By comparing before and after, it can be clearly observed that the arch dorsal elevation of S-T segment (red arrow). (b) After 14 d operation, the top row showed the normal rat hearts, and the bottom row showed the model rat hearts (left: Masson staining, right: HE staining). The myocardium of the left ventricle in the model group was atrophied and almost completely replaced by fibrous tissue, and a large number of blue collagen fibers were visible, while there was no change in the normal group.

**
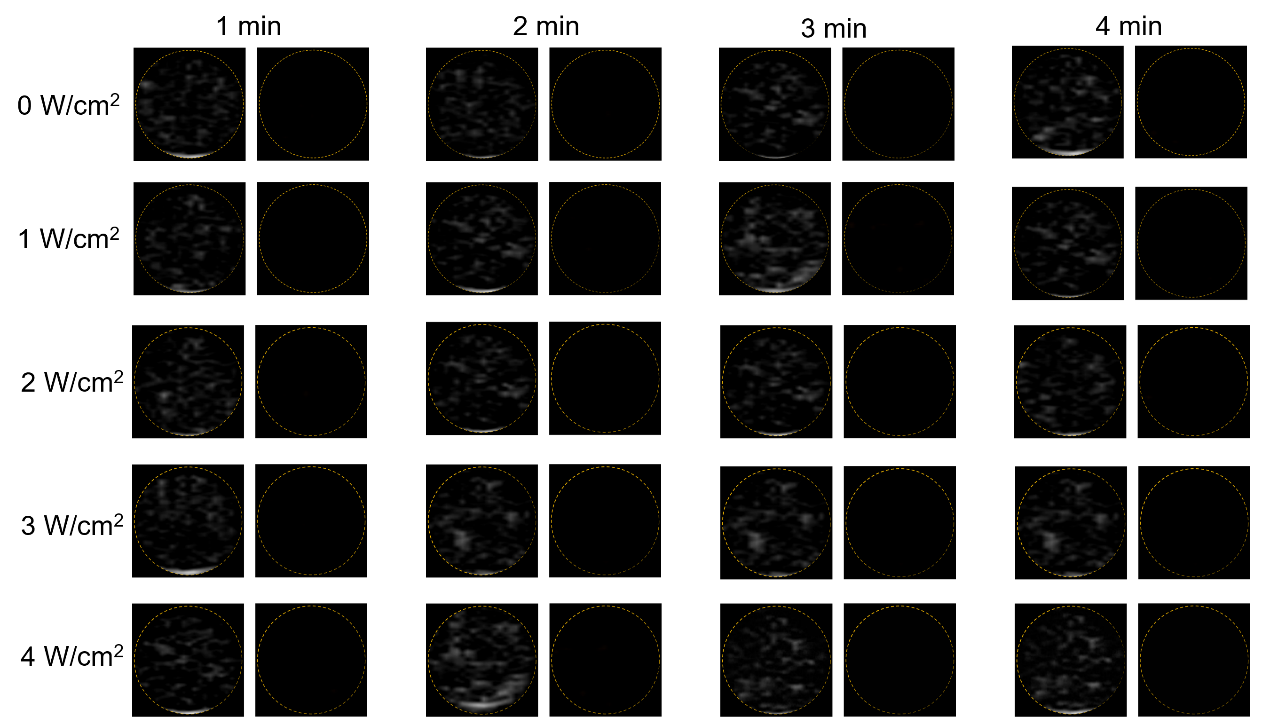
Figure S7.** ADV and US imaging of IMTP-Fe_3_O_4_ NPs at different intensities of LIFU irritation and different time in vitro. Echo intensity did not change in all cases.

**
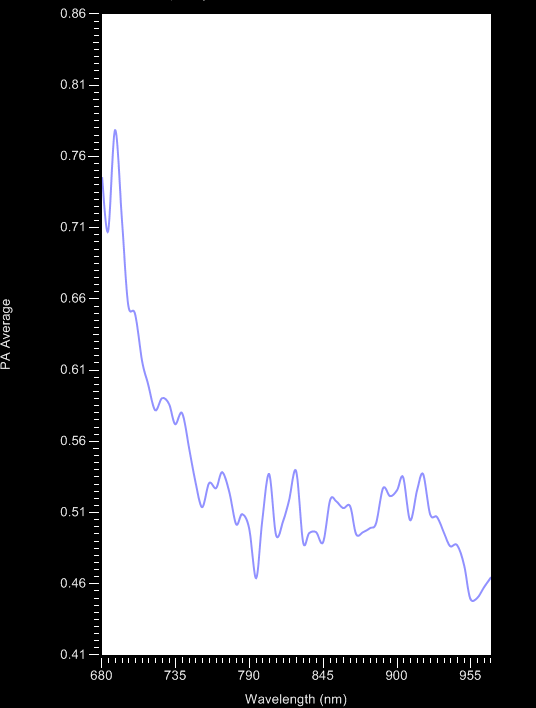
Figure S8.** The PA signal changes of IMTP-Fe_3_O_4_-PFH NPs irradiated by a laser at full spectrum ranging from 680 to 950 nm, and the peak appeared at 690 nm.
