# Supplementary material for: A non-invasive nanoparticles for multimodal imaging of ischemic myocardium in rats
Source: J Nanobiotechnology. 2021 Mar 22;19:82. doi: 10.1186/s12951-021-00822-7 (PMC7986298; doi:10.1186/s12951-021-00822-7)
Supplement: Supplementary file 2 — Additional file 2: Movie S1. Echocardiography before operation in rats. Movie S2. Echocardiography after operation in model rats. [file 12951_2021_822_MOESM2_ESM.zip › Additional file 2/Additional file 2.docx]

**Additional file 2**

# A Non-Invasive Nanoparticles For Multimodal Imaging Of Ischemic Myocardium In Rats

Xiajing Chen^1,2^, Yanan Zhang^1,2^, Hui Zhang^1,2^, Liang Zhang^3^, Lingjuan Liu^1,2^, Yang Cao^3^, Haitao Ran^3^, Jie Tian^1,2,*^

^1^Department of Cardiology; Ministry of Education Key Laboratory of Child Development and Disorders; National Clinical Research Center for Child Health and Disorders (Chongqing); China International Science and Technology Cooperation base of Child development and Critical Disorders; Children’s Hospital of Chongqing Medical University; Chongqing 400014, People’s Republic of China;

^2^Chongqing Key Laboratory of Pediatrics, Children’s Hospital of Chongqing Medical University, Chongqing,400014, People’s Republic of China;

^3^Chongqing Key Laboratory of Ultrasound Molecular Imaging & Department of Ultrasound, the Second Affiliated Hospital of Chongqing Medical University, Chongqing 400010, People’s Republic of China

*Corresponding Author: Jie Tian. Email: [jietian@cqmu.edu.cn](mailto:jietian@cqmu.edu.cn)

**Supplementary movie caption**

**Movie S1.** Echocardiography before operation in rats. Based on the distribution of coronary arteries and the innervation of the ventricular wall in rats, a short-axis left ventricular section was selected for observation. The movie illustrated that before operation, the myocardial contraction of the rats was strong, and the motion of the anterior and posterior walls was powerful and coordinated**.**

**Movie S2.** Echocardiography after operation in model rats. After ligation of the LAD coronary artery, the anterior wall motion of the left ventricle was seen to be reduced and the degree of systole-diastole was limited in the movie, demonstrating a successful model.
