# Supplementary material for: Correction: A non‑invasive nanoparticles for multimodal imaging of ischemic myocardium in rats
Source: J Nanobiotechnology. 2022 Aug 23;20:382. doi: 10.1186/s12951-022-01588-2 (PMC9400281; doi:10.1186/s12951-022-01588-2)
Supplement: Supplementary file 1 — Additional file 1: Figure S7. ADV and US imaging of IMTP-Fe3O4 NPs at different intensities of LIFU irritation and different time in vitro. Echo intensity did not change in all cases. [file 12951_2022_1588_MOESM1_ESM.docx]

**
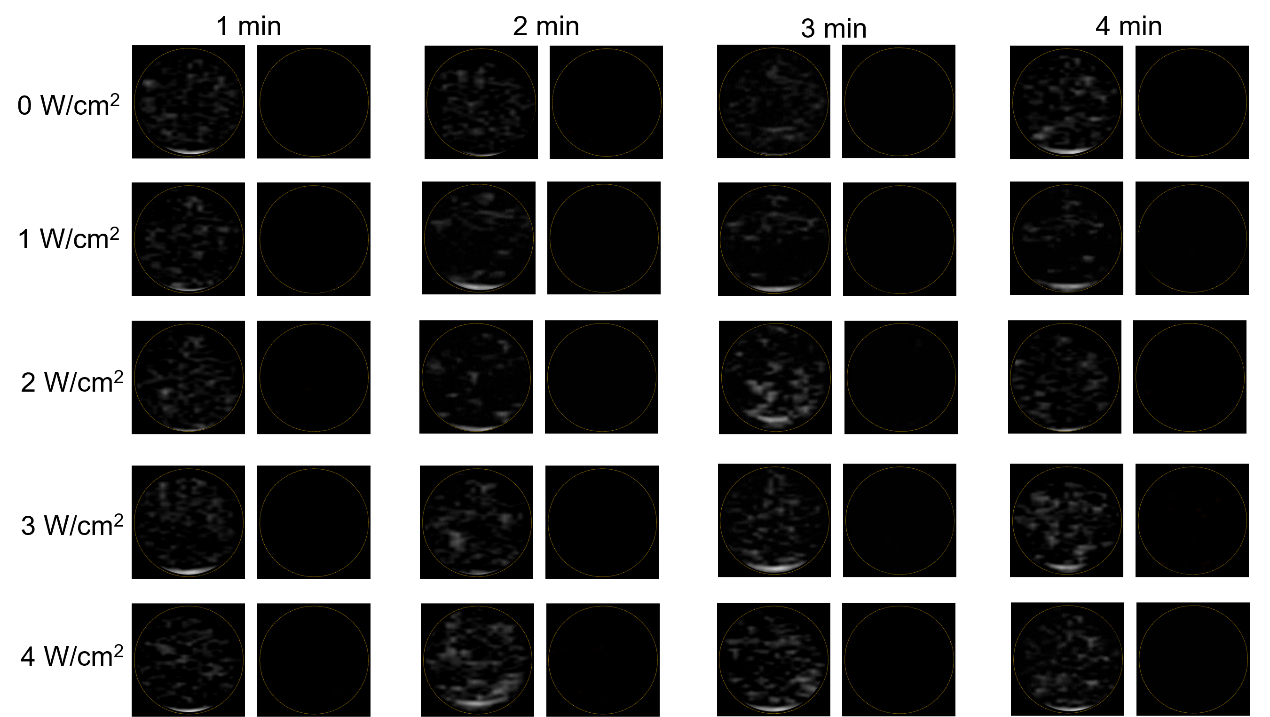
**

**Figure S7.** ADV and US imaging of IMTP-Fe_3_O_4_ NPs at different intensities of LIFU irritation and different time in vitro. Echo intensity did not change in all cases
